# Global translational and metabolic remodeling during iron deprivation in *Toxoplasma gondii*

**DOI:** 10.1128/mbio.03788-25

**Published:** 2026-04-02

**Authors:** Jack C. Hanna, Shikha Shikha, Megan A. Sloan, Clare R. Harding

**Affiliations:** 1 Glasgow Centre for Parasitology, School of Infection and Immunity, University of Glasgow 3526, Glasgow, United Kingdom; UT Southwestern Medical Center, Dallas, Texas, USA

**Keywords:** *Toxoplasma gondii*, iron regulation, proteomics, iron metabolism, carbon metabolism, mitochondrial metabolism, translation

## Abstract

Iron is required to support essential cellular processes. Due to diverse and dynamic host environments, the obligate intracellular parasite *Toxoplasma gondii* must adapt to iron-limited conditions. To investigate the adaptations critical to parasite survival under these conditions, we conducted proteomic and metabolomic profiling of *Toxoplasma* cultured in iron-depleted conditions. We find that iron depletion results in remodeling of the parasite proteome and triggers swift translational repression, prior to decreases in the key translational factor ABCE1. In the context of repressed translation, we also observe a significant rewiring of energy metabolism. Iron-depleted *Toxoplasma* have altered mitochondrial morphology and a profound reduction in mitochondrial respiration. Untargeted metabolomics revealed changes in central carbon metabolism, with the accumulation of intermediates of glycolysis and the tricarboxylic acid (TCA) cycle. Stable isotope labeling revealed that iron deprivation leads to a fundamental disconnect between these pathways, with reduced incorporation of glucose-derived carbon into cellular macromolecules and disruption of the TCA cycle. Instead, iron-deprived parasites continued to take up glucose and maintain glycolysis for energy generation. Limiting glucose availability, either in culture media or by genetic ablation of glucose uptake, caused a significant increase in sensitivity to iron restriction. Conversely, the limitation of mitochondrially metabolized glutamine improved parasite fitness in iron-depleted conditions. Together, our results establish iron as a key regulator of parasite translation and metabolic flexibility and demonstrate an increased reliance on glycolysis for energy generation and survival under acute iron deprivation.

**IMPORTANCE:**

This study determines the effects of iron deprivation on the parasite *Toxoplasma gondii*. Using proteomics and metabolomics, we reveal iron as a novel regulator of both protein translation and energy metabolism in *Toxoplasma,* underpinning the importance of this nutrient for essential cellular processes. We find that iron depletion introduces a metabolic bottleneck, whereby parasites become dependent on glucose as their major carbon source. By modulating the parasite’s metabolism by altering carbon source availability, we identify nutrient conditions that improve parasite survival under iron restriction. These data reveal a key role for adaptive plasticity of *Toxoplasma* central carbon metabolism to drive survival under iron-limited conditions. Understanding the interactions between parasite nutrient availability and metabolism allows us both to map the metabolic flexibility of these parasites and identify potential vulnerabilities.

## INTRODUCTION

Affecting approximately one-third of the global population, *Toxoplasma gondii* poses a persistent threat to human and animal health worldwide (1), and as an apicomplexan parasite, *Toxoplasma* is related to other parasites of significant human and veterinary importance, including *Plasmodium* (causative agent of malaria) and *Cryptosporidium* (causative agent of severe diarrheal disease). As an obligate intracellular parasite, *T. gondii* acquires essential nutrients from its host cell. These scavenged nutrients include carbon sources (e.g., glucose and glutamine), lipids, amino acids, and vitamin cofactors (2–5). Unlike other members of its phylum, however, *T. gondii* demonstrates an unusually broad host cell range, able to infect all warm-blooded species and any nucleated cell type. This promiscuity is key to the success of the parasite; however, the range of potential host environments necessitates flexibility in nutrient uptake and parasite metabolism (6–8).

Iron is an essential nutrient for eukaryotic cells, where it has been shown to be required for respiration, DNA replication, translation, and metabolism (9). Underlining this importance, diverse cells have established mechanisms for surviving iron starvation, although with distinct regulatory processes. These mechanisms are often characterized by translational inhibition (10, 11) and a metabolic switch away from oxidative phosphorylation toward glycolysis (12–14). As mitochondrial respiration requires significant proportions of cellular iron stores (15), this metabolic switch allows the cells to maintain energy generation under iron starvation. The proteomic remodeling and metabolic switch under iron depletion have significant functional consequences on cells, for example, in macrophages, iron starvation-induced metabolic changes reduce inflammation (12). Due to its central importance, mammals restrict iron availability to pathogens (16–18) through a range of mechanisms, including systemic induction of the hormone hepcidin, which restricts iron availability (19) and is likely upregulated in *Toxoplasma* infection (20).

In *Toxoplasma*, iron is incorporated into a heme biosynthesis pathway (21) and three distinct iron-sulfur (Fe-S) cluster biogenesis pathways (22, 23). These cofactors are essential components of proteins of mitochondrial metabolism and the electron transport chain (ETC). Our previous work has identified widespread transcriptional changes upon acute iron deprivation (24); however, changes in transcript abundance do not always correlate with functional adaptations, especially under stress (25), which concealed any potential parasite adaptation to low iron.

To investigate the functional response of *Toxoplasma*, we combined global proteomics and metabolomics to assess how the parasite responded to acute iron depletion. We find that iron depletion leads to rapid translational repression, accompanied by extensive proteomic remodeling. We assess the kinetics of repression and find that translational repression precedes a drop in the abundance of ABCE1, a key iron-regulated component of effective translation. The proteomic changes were accompanied by a collapse in mitochondrial oxidative phosphorylation and significant metabolic changes in the cytosol and mitochondria. Stable isotope labeling revealed changes in flux through central carbon metabolism in iron-deprived parasites. Interestingly, we find that carbon source availability modulates the parasite’s sensitivity to iron deprivation; inhibition of glycolysis led to increased sensitivity, while glutamine restriction, which likely promotes energy generation through glycolysis, leads to increased growth under low iron. Our results demonstrate that *Toxoplasma* actively responds to iron deprivation by remodeling its proteome, shifting energy generation to glycolysis and demonstrating the flexibility and adaptability of these intracellular parasites to nutrient stress.

## RESULTS

### Iron deprivation leads to remodeling of the proteome

**Fig 1:**
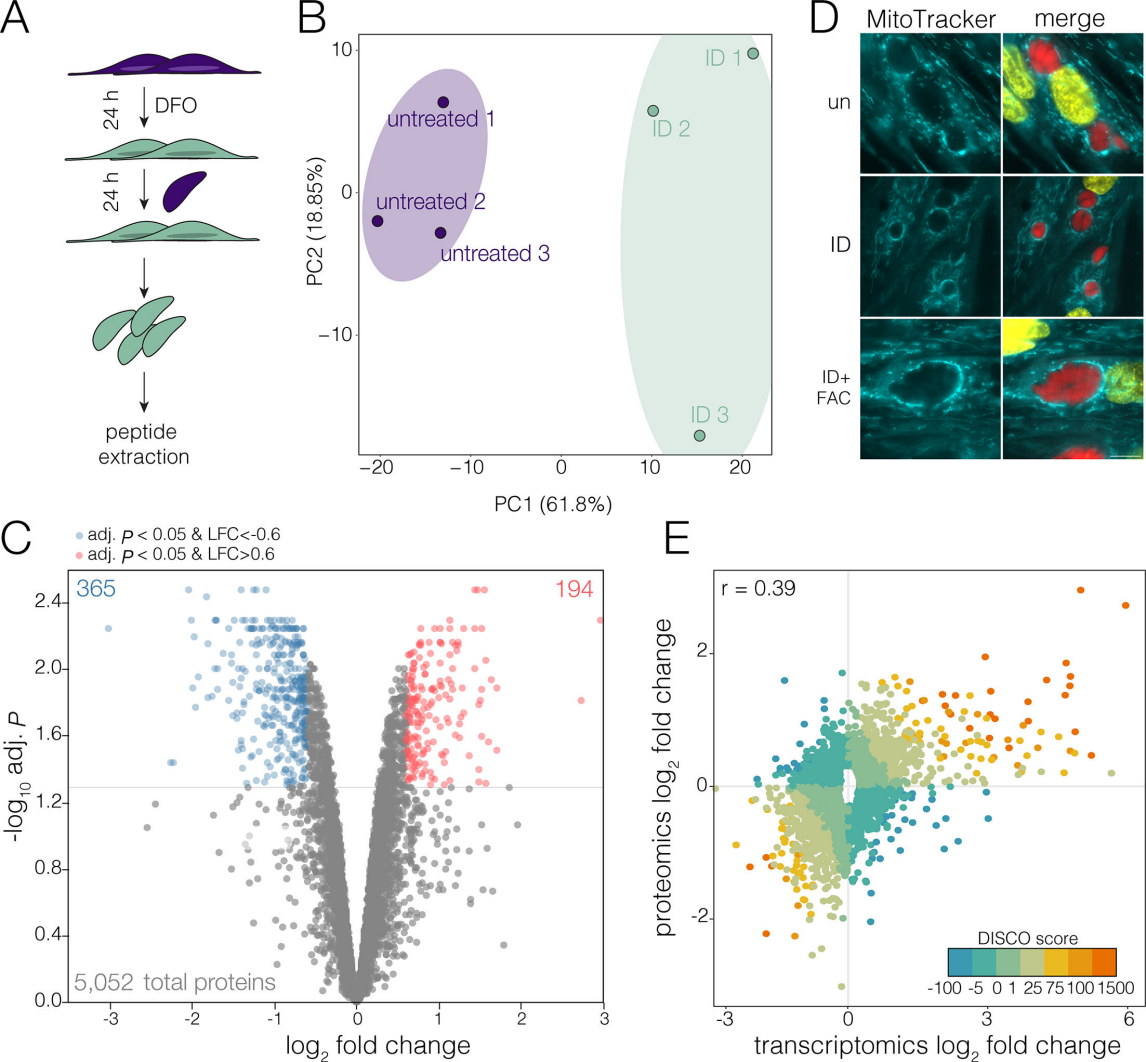
Iron deprivation leads to remodeling of the proteome. (**A**) Schematic showing the sample collection workflow. Parasites were collected after 24 h of infection on untreated host cells or cells that were pretreated with 100 µM DFO for 24 h prior to infection (ID). (**B**) Principal components analysis (PCA) of untreated and iron-depleted (ID) samples showing variation between biological replicates and conditions. Ellipses represent the 95% confidence region for each grouping. (**C**) Volcano plot of differential protein abundance analysis, with log_10_ adjusted *P*-value plotted against the log_2_ fold change (ID/untreated). Significant (adj. *P* < 0.05, log_2_ fold change (L2FC) <−0.6 or >0.6) changing proteins are indicated in red and blue. (**D**) Live imaging of host mitochondrial association (HMA) by MitoTracker (cyan) in host cells cultured as above. For ID+FAC, host cells were pretreated with 100 µM DFO and 200 µM ferric ammonium citrate (FAC). MitoTracker staining localized adjacent to parasite vacuoles (red). DNA is shown in yellow. Scale bar: 10 µm. (**E**) Plot of proteomics log_2_ fold change against transcriptomics log_2_ fold change from a previous study (24). Only genes that were significantly different in at least one data set (adj.*P* < 0.05) were included in the analysis. A linear function was plotted (line not shown) to obtain a correlation coefficient (*R*) of 0.39; points were colored by DISCO score. Genes that correlate positively between data sets have more positive (orange) DISCO scores, while those that are not correlated or negatively correlated have smaller or negative (blue) DISCO scores.

To investigate the impact of acute iron deprivation on the *Toxoplasma* proteome, we cultured *Toxoplasma* for 24 h in the presence of the iron chelator deferoxamine (DFO) in previously iron-starved host cells (pretreated for 24 h with DFO) and quantified the changes in parasite protein abundance by quantitative mass spectrometry (Fig. 1A). Our analysis included 5,052 proteins, detected in at least two out of three replicates. PCA analysis showed good grouping of samples between biological experiments, with clear separation between treatment conditions (Fig. 1B). Iron deprivation led to significant remodeling of the parasite proteome (Fig. 1C; Table S1), with 194 proteins significantly upregulated (adjusted *P* < 0.05, log_2_ fold change [LFC] > 0.6) and 365 significantly downregulated (adj. *P* < 0.05, LFC < −0.6).

*Toxoplasma* encodes 70 predicted Fe-S-containing proteins (26), which gain Fe-S clusters through cytosolic, apicoplast, or mitochondria-localized pathways (22, 27). Disruption of Fe-S cluster biosynthesis pathways can result in destabilization of the dependent Fe-S proteins (22, 27, 28). We detect 66 of these predicted Fe-S proteins in our data set, with 10 significantly changing in abundance in iron-deprived parasites (Fig. S1A). Interestingly, we did not see the depletion of Fe-S proteins dependent on mitochondrial or apicoplast-localized pathways; however, of the eight Fe-S proteins known to require the cytosolic Fe-S pathway (28), five were depleted in our data set (Table 1). These data suggest either that DFO is more capable of accessing cytosolic proteins over organellar restricted proteins, or that cytosolic Fe-S proteins may be more labile upon iron deprivation.

**TABLE 1:** Cytosolic and nuclear Fe-S proteins (identified in a previous study (26), which are downregulated in low iron conditions

Gene ID	Name	Log_2_ fold change (this study)	Localization (29)	Function
TGGT1_258030	POLD1	−1.71	Nucleolus	Subunit of DNA polymerase delta complex
TGGT1_238190	POLR1C	−0.42	Nucleus non-chromatin	Subunit of DNA-directed RNA polymerases I and III
TGGT1_297840	PRIM2	−1.26	No data	Subunit of DNA primase and DNA polymerase alpha complex
TGGT1_216790	ABCE1	−0.66	Nucleus non-chromatin	Ribosome recycling factor
TGGT1_267390	POLR1C	−0.68	Nucleus non-chromatin	Subunit of DNA-directed RNA polymerases I and III
TGGT1_319860	POLE1	−1.07	No data	Subunit of DNA polymerase epsilon complex

Previously, iron starvation in *Toxoplasma* was shown to lead to an increase in lipid bodies (23), and we see an upregulation of the 2-acylglycerol O-acyltransferase (TGGT1_226370) which plays a crucial role in the formation of triglycerides that are stored within lipid droplets. *Toxoplasma* encodes the pathways for both the synthesis and scavenging of lipids. In our data, we see a decreased abundance of ATP citrate lyase (ACL; TGGT1_223840), which is used to synthesize acetyl-CoA from TCA-derived citrate, and a concurrent upregulation of acetyl-CoA synthetase (ACS; TGGT1_266640). ACS was previously shown to generate cytosolic acetyl-CoA from host-derived acetate for fatty acid elongation (30). Previous quantification of lipid species also found an increased sphingomyelin abundance upon iron starvation (23). However, we observed downregulation of both the serine palmitoyltransferase SPT1 (TGGT1_290980) and the ceramide synthase CERS2 (TGGT1_316450), both required for the *de novo* synthesis of ceramides and sphingomyelin. Previous data have shown that parasites preferentially scavenge sphingomyelins under nutrient-replete conditions (31). An increase in sphingomyelin, despite the downregulation of synthesis enzymes, may therefore reflect increased sphingolipid scavenging by the parasite.

Taken together, these data indicate an increase in scavenging and a decrease in the synthesis of fatty acid building blocks, suggesting increased storage of fatty acids within lipid droplets upon iron deprivation.

We did not detect changes in the abundance of the major iron and zinc-uptake transporter (ZFT) or in the vacuolar iron transporter (VIT) (32, 33); however, the predicted mitochondrial iron transporter (MIT; TGGT1_277090) did increase in abundance. Of the 194 proteins significantly upregulated, 70 of these were predicted to localize to the dense granules (DG). These proteins are typically secreted from the parasite to engineer a permissive environment for parasite growth (34). One potential hypothesis for the accumulation of DG proteins in the parasite is reduced secretion under iron deprivation. To determine if dense granule secretion occurred in iron-deprived cells, we examined host mitochondrial association (HMA) to the parasitophorous vacuole (PV). HMA is driven by the secretion of MAF1b, an isoform of a dense granule protein that specifically recruits host mitochondria (35). However, even in iron-deprived parasites, we saw a strong association of the host mitochondria with the PV (Fig. 1D). We also examined the localization of the DG protein Gra7 (36) and observed robust Gra7 secretion to the PV in iron-deprived parasites (Fig. S1B), demonstrating that iron-deprived parasites are capable of secreting DGs into the host cell.

Previously, we performed RNA-seq on iron-deprived parasites under identical conditions (24). To determine how changes in transcript abundance correlate with changes in the proteome, we correlated the fold changes in transcript or protein abundance after 24 h of iron deprivation (Fig. 1E; Table S2). Points were colored by discordance/concordance score (DISCO) based on the local FDR-corrected *q*-value for each L2FC (37). We find that changes in protein abundance show limited correlation (Pearson’s correlation *r* = 0.39) with transcript abundance (Fig. 1E), and 1,060 genes (34% of significantly changed genes) had a DISCO score of <0, suggesting high discordance between the approaches. Previous studies across organisms have seen disconnects between the transcriptome and proteome, especially under stress (38, 39). Interestingly, in our transcriptomic data, we see a general upregulation of transcripts, while at the proteome level, we see an overall downregulation of protein abundance. This discordance could be created by changes in translation under iron deprivation.

### Iron depletion leads to translational repression

**Fig 2:**
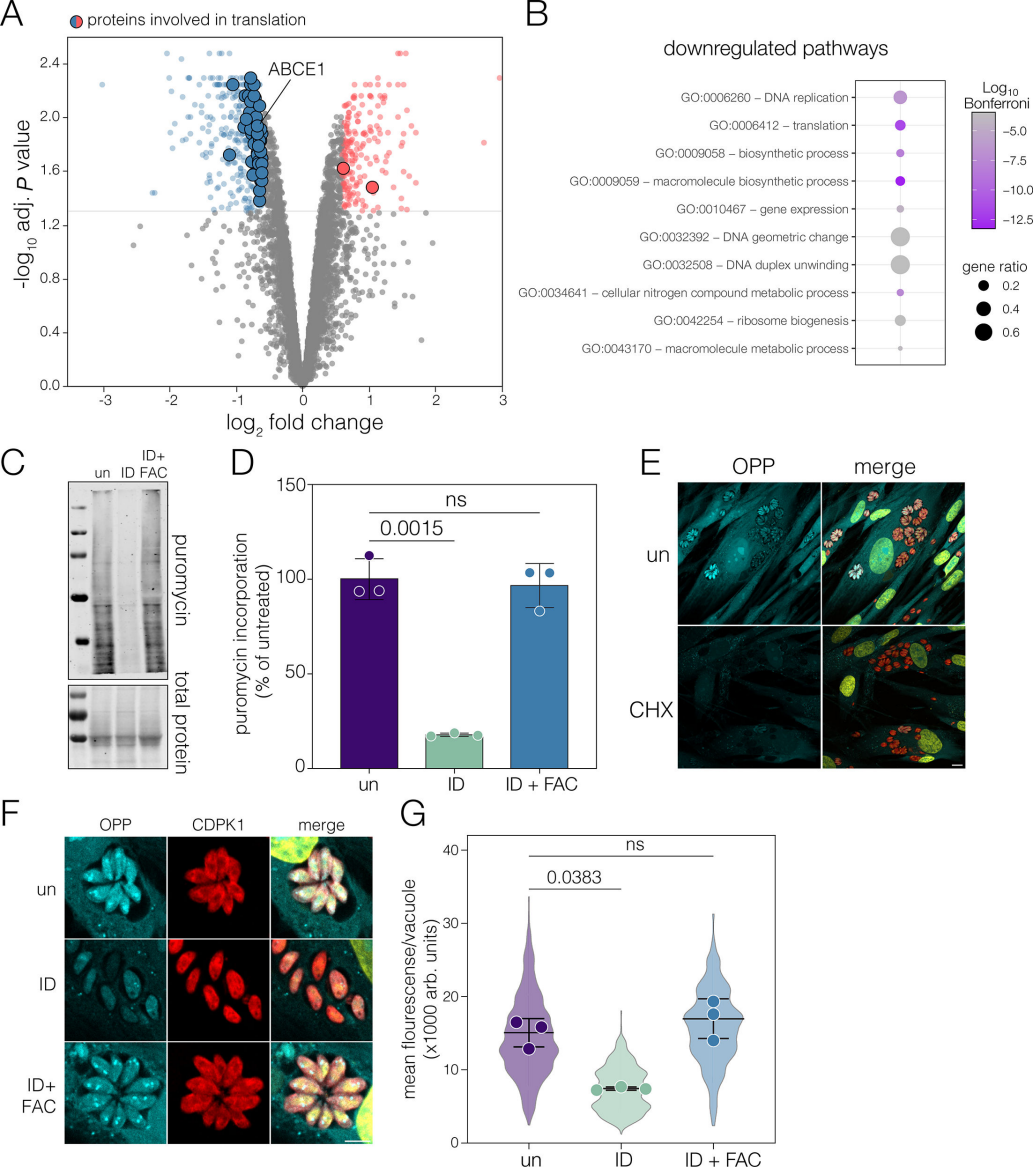
Iron-depleted *Toxoplasma* downregulates translation. (**A**) Volcano plot from Fig. 1C with proteins significantly (adj. *P* < 0.05, L2FC < 0.6) changed upon ID and involved in translation (using GO-term [GO: 0006412]) highlighted. (**B**) Dot plot of the top 10 statistically significant GO terms from downregulated proteins, and the size represents the proportion of genes annotated with each GO term. The color intensity represents the Bonferroni-corrected *P*-value. (**C**) Parasites were treated as indicated and incubated with puromycin for 30 min. Total protein visualized by Ponceau S staining. Puromycilated peptides were detected using an anti-puromycin antibody. (**D**) Quantification of puromycin incorporation, with intensity normalized to total protein and untreated mean. Each point represents a biological replicate; bar represents the mean ± SD. *P*-values from Brown-Forsythe and Welch ANOVA. (**E**) Immunofluorescence images of *Toxoplasma* vacuoles labeled with o-propargyl puromycin (OPP) (cyan), with and without co-incubation of cycloheximide (CHX). CDPK1 is used as parasite cytoplasmic control (red). DNA is shown in yellow. Scale bar is 10 µm. (**F**) Immunofluorescence imaging of OPP in untreated (top) 24 h ID (middle) and ID + FAC (bottom) *Toxoplasma*. DNA is visualized in yellow. Scale bar: 5 µm. (**G**) Quantification of OPP incorporation/vacuole. Each point represents a biological replicate; bar represents the mean ± SD. *P*-values from one-way ANOVA with Tukey’s multiple comparison correction. Violins represent the distribution of all vacuoles measured from each condition (*N* = 537, 573, and 476, respectively). ns, not significant.

Of the 365 proteins downregulated, 47 are involved in translation (Fig. 2A), and translation is a highly enriched GO term among proteins with decreased abundance (Fig. 2B). To determine the functional consequence of iron deprivation, we quantified total protein translation under iron deprivation using puromycin incorporation (28). Puromycin is an aminonucleoside which is readily incorporated into nascent peptides during translation, allowing the detection of puromycilated peptides with specific antibodies. After 24 h of iron deprivation, we saw a significant (*P* value = 0.0015, one-way ANOVA) decrease in global puromycin incorporation in the parasites after 30 min of treatment, when normalized to total protein (Fig. 2C and D), demonstrating an inhibition of translation. To confirm this inhibition was due specifically to iron depletion, addition of exogenous iron (200 µM ferric ammonium citrate [FAC]) along with DFO led to no change in translation (Fig. 2D).

Puromycin will also be incorporated by the host cells, which will also be impacted by iron deprivation (11), potentially complicating our results. To validate the effect on the parasites, we used an analog of puromycin, O-propargyl puromycin (OPP) (40), which allows for the covalent attachment of fluorophores to puromycilated peptides by click chemistry. OPP incorporation was seen in both the host and parasites and was heterogeneous between vacuoles, potentially reflecting the asynchronous cell cycle of *Toxoplasma* vacuoles (Fig. 2E) (41). To confirm the specificity of OPP, incorporation could be effectively blocked by cycloheximide (CHX) (Fig. 2E; Fig. S2A), an inhibitor of cytosolic translation. Quantifying OPP incorporation demonstrated a significantly (*P* = 0.038, one-way ANOVA) reduced signal in iron-deprived vacuoles, which was not seen upon DFO and iron co-treatment (Fig. 2F and G).

These data confirm that iron deprivation induces global translational repression in *Toxoplasma*.

### Translational repression is rapid and precedes ABCE1 depletion

Of the Fe-S proteins found to be significantly downregulated upon iron deprivation (Table 1), ABCE1 has a well-documented role in ribosome recycling during translation (42) and is iron-regulated in mammalian cells (42, 43). There are conflicting data on the result of depletion of ABCE1 on translation in mammalian cells, with some studies showing that this is sufficient to repress translation (44, 45), while others see only a minor effect (43).

**Fig 3:**
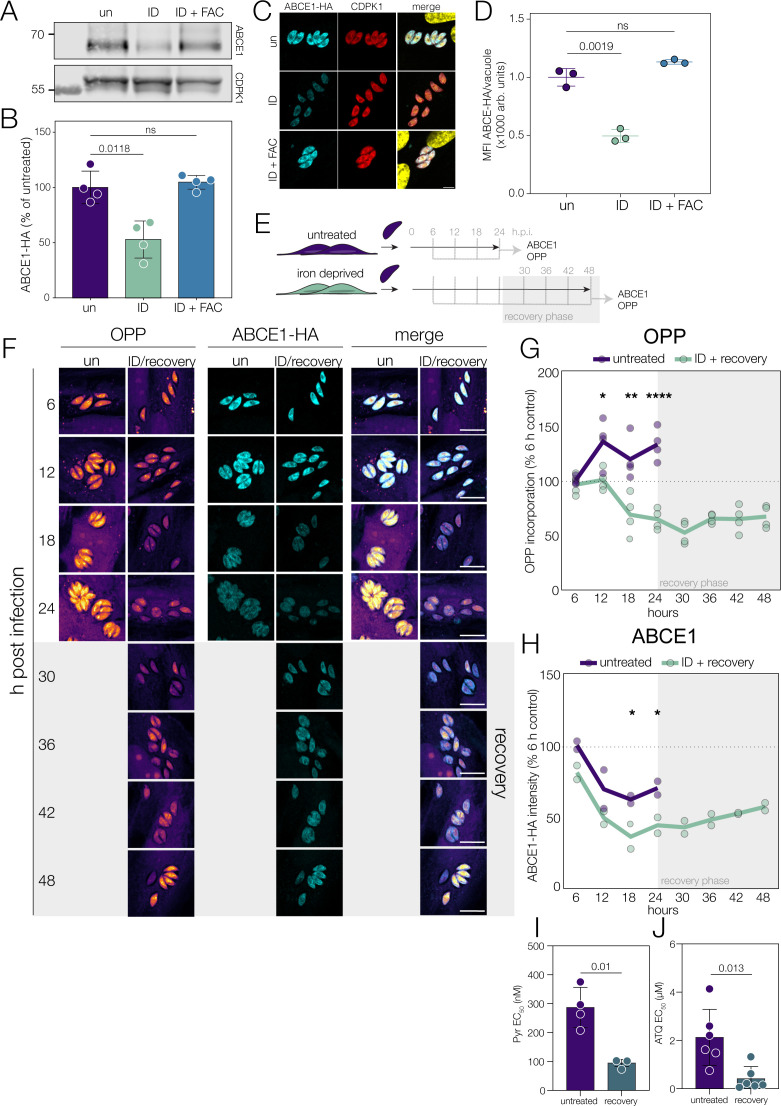
Translational repression is rapid and precedes ABCE1 depletion. (**A**) Immunoblot of ABCE1-HA lysates from untreated, ID, or ID + FAC parasites. CDPK1 is included as a loading control. (**B**) ABCE1-HA abundance was quantified and normalized to CDPK1 abundance and untreated parasites; points indicate biological replicates, and bars represent mean ± SD. *P*-values were determined using one-way ANOVA with Dunnett’s correction for multiple comparisons. (**C**) Immunofluorescence of cytoplasmic ABCE1-HA (cyan) parasites grown as above; CDPK1 (red) included as a cytoplasmic marker, and DNA is shown in the merge (yellow). Scale bar: 5 µm. (**D**) ABCE1-HA signal was quantified from IFA (*n* = 3); bars represent mean ± SD. *P* values were obtained using Brown-Forsythe and Welch ANOVA. (**E**) Schematic depicting the translation imaging time course. Untreated and ID parasites were fixed and imaged every 6 h for 24 h. ID parasites allowed to recover in iron-replete media were fixed for a further 24 h (recovery). (**F**) ABCE1-HA abundance and OPP incorporation through ID and recovery. Scale bar 10 µm. Mean (lines) of OPP (**G**) and ABCE1-HA signal (**H**) plotted relative to untreated 6 h. *P* values from comparing ID with untreated, two-way ANOVA with Tukey correction, * *P* < 0.03, ** *P* < 0.003, *** *P* < 0.0001. (**I**) Average EC_50_ of pyrimethamine and atovaquone (**J**) against untreated parasites and parasites recovering from iron depletion. Points indicate biological replicates, and bars represent mean ± SD. *P* values were obtained from two-tailed *t-*test.

In our data set, ABCE1 is significantly depleted (Fig. S2B) (log_2_FC = −0.66, FDR-adjusted *P =* 0.012). To confirm this, we used an endogenously ABCE1-HA-tagged line (28) and saw decreased ABCE1-HA abundance (*P* = 0.0118, one-way ANOVA) under low iron by western blot (Fig. 3A and B), normalized to CDPK1, a highly abundant protein unchanged in proteomics (Table S1). We confirmed by immunofluorescence assay and saw a significant and reproducible (*P* = 0.0019, one-way ANOVA) drop in ABCE1-HA signal upon iron deprivation (Fig. 3C and D).

To examine the kinetics of ABCE1 depletion in the context of translational repression, we performed a time course of iron deprivation and subsequent recovery when parasites were given iron-replete media after 24 h of starvation (Fig. 3E). At each time point, we quantified vacuolar area, OPP incorporation (as a measure of translation), and ABCE1-HA abundance in each vacuole. Mean vacuolar area did not increase during iron depletion, as expected (Fig. S2C). However, replacement with iron-replete media after 24 h of iron depletion did lead to a significant (*P* = 0.03, one-way ANOVA) increase in vacuolar area from 12 h post-recovery, indicating that parasites were able to reinitiate replication upon recovery and demonstrating that the effects of acute iron depletion are largely reversible.

We also quantified host cell OPP incorporation throughout the experiment. Host translation was notably lower than that of parasites at all points, and iron depletion significantly reduced host translation (*P* < 0.0001, two-way ANOVA with Tukey’s correction) at each time point, as previously described (11). Host translation recovered by 36 h post-treatment (12 h post-recovery) with no significant change from untreated host cells (Fig. S2D)

In the parasite, the levels of OPP incorporation are consistent between vacuoles at early time points but become more diverse after 6 h, potentially reflecting the progressive loss of synchronous cell division (Fig. S2E). Upon iron depletion, the first significant (*P* = 0.039, two-way ANOVA with Tukey’s correction) difference in translation appears at 12 h post-infection (Fig. 3F and G), as iron-deprived parasites no longer increase translation. After 12 h of iron depletion, the levels of OPP incorporation progressively decrease (Fig. 3F and G). However, upon recovery, the mean OPP incorporation significantly (*P* = 0.034, two-way ANOVA with Tukey’s correction) increases (Fig. S2F), demonstrating the recovery of translation under iron-replete conditions.

Recent single-cell transcriptomics identified ABCE1 as cell cycle regulated, with a wave of transcription occurring in the G1-S phase (46). We see a peak of ABCE1-HA abundance at 6 h post-infection, which then drops in the untreated cells (Fig. 3F and H). There is no significant change in ABCE1-HA abundance under iron depletion until 18 h (*P* = 0.03, two-way ANOVA with Tukey’s correction), despite the drop in translation. Upon recovery, there is a gradual (although not significant) increase in ABCE1 abundance over the course of the experiment (Fig. S2F).

The stall in translation, prior to ABCE1 abundance changes, and the failure of iron-replete parasites to recover ABCE1 levels suggest that loss of ABCE1 is not the major driver of translational repression in iron-depleted parasites, although it is possible that the activity of ABCE1 is altered prior to its drop in abundance.

Addition of iron to deprived parasites triggered a recovery of translation and replication. To determine if this recovery led to an increased reliance on key metabolic pathways, we iron-deprived parasites for 24 h, replaced with iron-replete media, and tested sensitivity to metabolic inhibition. Recovering parasites were significantly (*P* = 0.009, *t*-test) more sensitive to pyrimethamine, an inhibitor of folate biosynthesis required for nucleotide synthesis (Fig. 3I; Fig. S2G) and inhibition of the mitochondrial ETC by atovaquone (*P* = 0.013, *t*-test) (Fig. 3J; Fig. S2H). However, we saw no change in sensitivity upon treatment with the broad anti-parasitic dihydroartemisinin (Fig. S2I and J). This suggests that upon recovery from iron deprivation, parasites are more sensitive to further metabolic inhibition.

### Iron deprivation leads to alterations in mitochondrial metabolism

**Fig 4:**
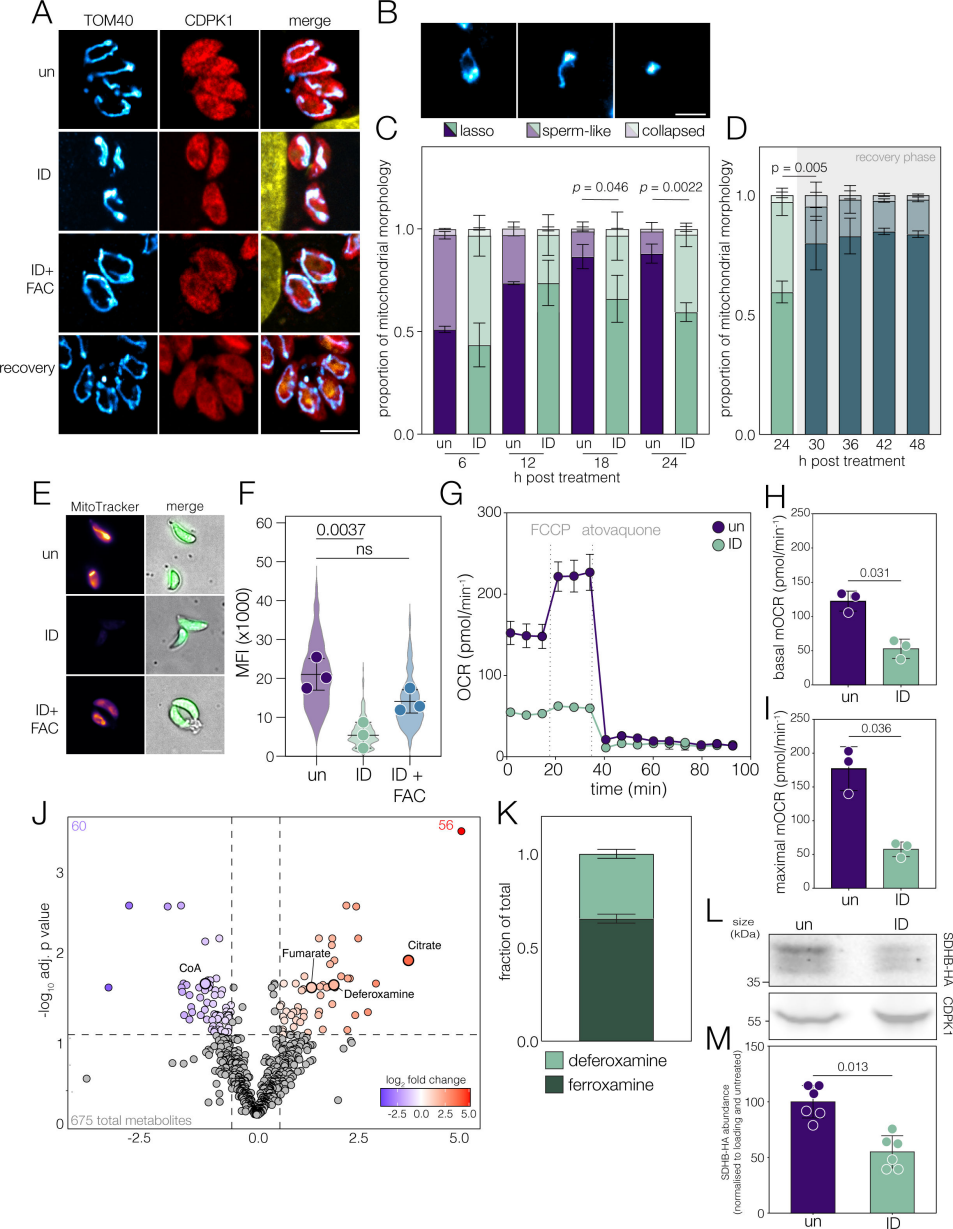
Iron deprivation leads to changes in mitochondrial metabolism. (**A**) Mitochondrial morphology is observed using the mitochondrial membrane marker TOM40, and parasite cytoplasm is labeled using anti-CDPK1 (red). Scale bar: 5 µm. (**B**) Classification of mitochondrial morphologies, based on a previous study (52). Scale bar: 5 µm. Quantification of mitochondrial morphology across untreated and ID (**C**) and recovery (**D**); 100 parasites quantified in each replicate, *N* = 2**,** *P*-value from two-way ANOVA with Tukey correction for multiple comparisons. (**E**) Representative live imaging of MitoTracker Deep Red staining in extracellular ΔKu80:mNeonGreen *Toxoplasma* grown in ID or ID + FAC conditions. Merged images show cytoplasmic mNeon and a polarized light image. Scale bar: 5 µm (**F**) Quantification of Mitotracker intensity across three biological replicates (points), overlaid on a violin plot showing the distribution of individual parasites measured (*N* = 294/262/305). *P* values from one-way ANOVA with Tukey correction for multiple comparisons. (**G**) Representative oxygen consumption rate (OCR) trace for untreated and ID parasites. Background OCR was subtracted to determine mitochondrial-associated oxygen consumption rate (mOCR). (**H**) Basal and (**I**) maximal mOCR were determined for both untreated and iron-depleted parasites after 24 h of infection. Each point represents a replicate (*n* = 3); bar represents mean ± SD. *P*-values are obtained from two-tailed paired *t*-tests. (**J**) Volcano plot showing identified metabolites log_10_ adjusted *P*-value (computed by FDR-adjusted *t*-tests) against corresponding log_2_ fold change (ID/untreated). Metabolites with adjusted *P* < 0.1 and a log_2_ fold change of >0.6 or <−0.6 are colored based on the magnitude of fold change, non-significant metabolites in gray. (**K**) Stacked bar graph showing the fraction of total iron chelator detected that is bound (ferroxamine) or unbound (deferoxamine) by iron. Normalized peak intensities for each were divided by their combined totals for each replicate. (**L and M**) Immunoblot of lysates from SDHB-HA parasites, untreated or ID; CDPK1 is included as a loading control. Points represent biological replicates (*n* = 6); bar represents mean ± SD. *P* values were obtained from a two-tailed paired *t*-test.

Beyond translation, iron is essential in the mitochondria where its incorporation into the proteins of the electron transport chain (ETC) is required for mitochondrial respiration and energy generation (47–49). *Toxoplasma* has a single dynamic mitochondrion, which has been shown to alter morphology upon stress (50), genetic ablation of key components (47, 51), and extracellular exposure (52). We examined parasite mitochondrial morphology at 24 h post-iron starvation and saw that mitochondria from iron-depleted parasites had lost the circular “lasso” shape observed in untreated parasites (Fig. 4A). This phenotype was fully reversible, as the mitochondrion reverted to normal after a further 24 h in iron-replete media (Fig. 4A). To understand the kinetics of mitochondrial morphology, we collected immunofluorescence images from untreated and iron-deprived parasites every 6 h for 24 h of infection and 24 h of recovery and assigned mitochondria into three previously established morphology classes (52) (Fig. 4B). At both 6 and 12 h post-infection, mitochondrial morphology is unchanged. However, at 18 h, iron depletion causes a significant decrease in the proportion of lasso mitochondria compared to untreated parasites (*P* = 0.046 at 18 h, *P* = 0.0022 at 24 h, two-way ANOVA) (Fig. 4C). Upon recovery, after 6 h of culture in iron replete, there was a significant increase in the number of lasso-shaped mitochondria (*P* = 0.005, two-way ANOVA), reflective of the proportions seen in untreated intracellular parasites (Fig. 4D).

As dynamic changes to morphology may indicate changes in function, we assessed mitochondrial membrane potential using MitoTracker, a dye that accumulates in mitochondria and fluoresces in the presence of an active proton gradient. We observed a significant (*P* = 0.0037, one-way ANOVA) decrease in MitoTracker signal in DFO-treated parasites (Fig. 4E and F), suggesting that iron deprivation leads to a loss of proton gradient in the parasites.

To gain an integrated view of cellular energy metabolism, we performed metabolic flux analysis using a Seahorse Analyzer (49, 53), which allows quantification of the mitochondrial oxygen consumption rate (mOCR). We find that iron deprivation leads to a significant drop both in basal mOCR (Fig. 4G and H; *P* = 0.031*,* paired *t*-test) and maximal mOCR (Fig. 4I; *P* = 0.036, paired *t*-test), demonstrating a significant defect in mitochondrial respiratory capacity upon iron starvation.

These data demonstrate that iron deprivation leads to significant defects in mitochondrial polarization and respiration.

### Iron deprivation leads to global metabolic changes

Mitochondrial respiration is central to cellular metabolism; however, many metabolic processes beyond the mitochondrion rely on iron-bound enzymes. To understand the global effects of iron depletion on parasite metabolism, we performed untargeted metabolomics on iron-depleted parasites; 675 metabolites were annotated across four biological replicates, and the PCA plot (Fig. S3A) demonstrated good separation between experimental conditions. Overall, we identified 56 upregulated metabolites (adj. *P* < 0.1, log_2_ fold change [LFC] > 0.6) and 60 significantly downregulated (adj. *P* < 0.1, LFC < −0.6) (Fig. 4J; Fig. S3B; Table S3). Under iron deprivation, both deferoxamine and the iron-bound ferrioxamine were detectable; we find that approximately 65% of the chelator was iron-bound (Fig. 4K), demonstrating that DFO is in excess under these conditions. To identify the pathways of importance in our data set, we performed pathway analysis, incorporating topology; hence, pathways with closely associated metabolites are assigned a higher “pathway importance” score (Fig. S3C). Using this method, we highlighted alterations in the TCA cycle and the pentose phosphate pathway-demonstrating a link between iron and central carbon metabolismand significant changes in amino acid metabolism.

Iron depletion has previously been associated with perturbations to amino acid metabolism in other organisms (39, 54), although its role in *Toxoplasma* amino acid pathways has not been assessed. Of the metabolites with the largest increases in abundance, we identified 5-aminopentanoic acid and lysopine, both of which are lysine degradation derivatives (55), which suggests that protein turnover imbalance, potentially due to translational repression, may play a role.

We also see significant changes in fumarate and citrate (Fig. 4J), both metabolites of the TCA cycle, with a 14-fold increase in citrate abundance in iron-depleted parasites. Citrate in *Toxoplasma* is utilised both in the TCA cycle and is metabolized in the cytoplasm by ATP-citrate lyase (ACL) (TGGT1_223840). Our proteomics reveals a significant (L_2_FC −0.832, adj. *P* value = 0.035) decrease in ACL abundance in iron deprivation. In the mitochondrion, citrate is metabolized to iso-citrate by aconitase, a key Fe-S-containing enzyme, which, in mammalian systems, is tightly linked with iron homeostasis (56, 57). We have previously shown inhibition of aconitase activity in iron-deprived *Toxoplasma* (24); however, to confirm this, we tested the sensitivity of aconitase to chemical inhibition under low iron conditions (2, 58). We treated parasites with 20 µM DFO—predicted to inhibit parasite replication by around 20%—to limit iron but allow replication; 20 µM DFO-treated parasites become significantly (*P* < 0.0001, extra sum-of-squares F test) more sensitive to NaFAc (IC_50_ diff = −37.33 nM) (Fig. S4A), suggesting that iron restriction makes parasites more sensitive to aconitase inhibition, potentially because most of the enzyme has lost the Fe-S clusters required for activity (59).

Fumarate is increased 2.6-fold in iron deprivation and is generated by succinate dehydrogenase, which also plays a role in the ETC. To investigate this further, we examined the abundance of the key subunit, succinate dehydrogenase B (SDHB) (TGGT1_215280). Previously, we saw that knockdown of the major iron transporter ZFT led to decreased SDHB abundance (33). Using a parasite line expressing endogenously tagged SDHB (SDHB-HA) (60), we determined that SDHB was significantly (*P* = 0.013,paired *t*-test) downregulated under iron depletion (Fig. 4L and M). This appeared specific, as the related Fe-S-containing ETC subunit, Rieske-FLAG (61), did not change in abundance under low iron conditions (Fig. S4B and C), potentially indicating that these proteins differ in their stability under stress or suggesting targeted regulation of SDHB. Despite decreased SDHB abundance, we see fumarate accumulation. Fumarate is hydrated to malate by fumarate hydratase, a 4Fe-4S protein (TGGT1_267330) (62–64), which, in other organisms, has been shown to be inhibited by iron deprivation (65, 66). We suggest that in low iron, fumarate hydratase may lose activity (potentially due to Fe-S cluster loss), despite sustained protein abundance, leading to fumarate accumulation in low iron.

### Stable isotope labeling reveals disconnects between glycolysis and TCA cycle

**Fig 5:**
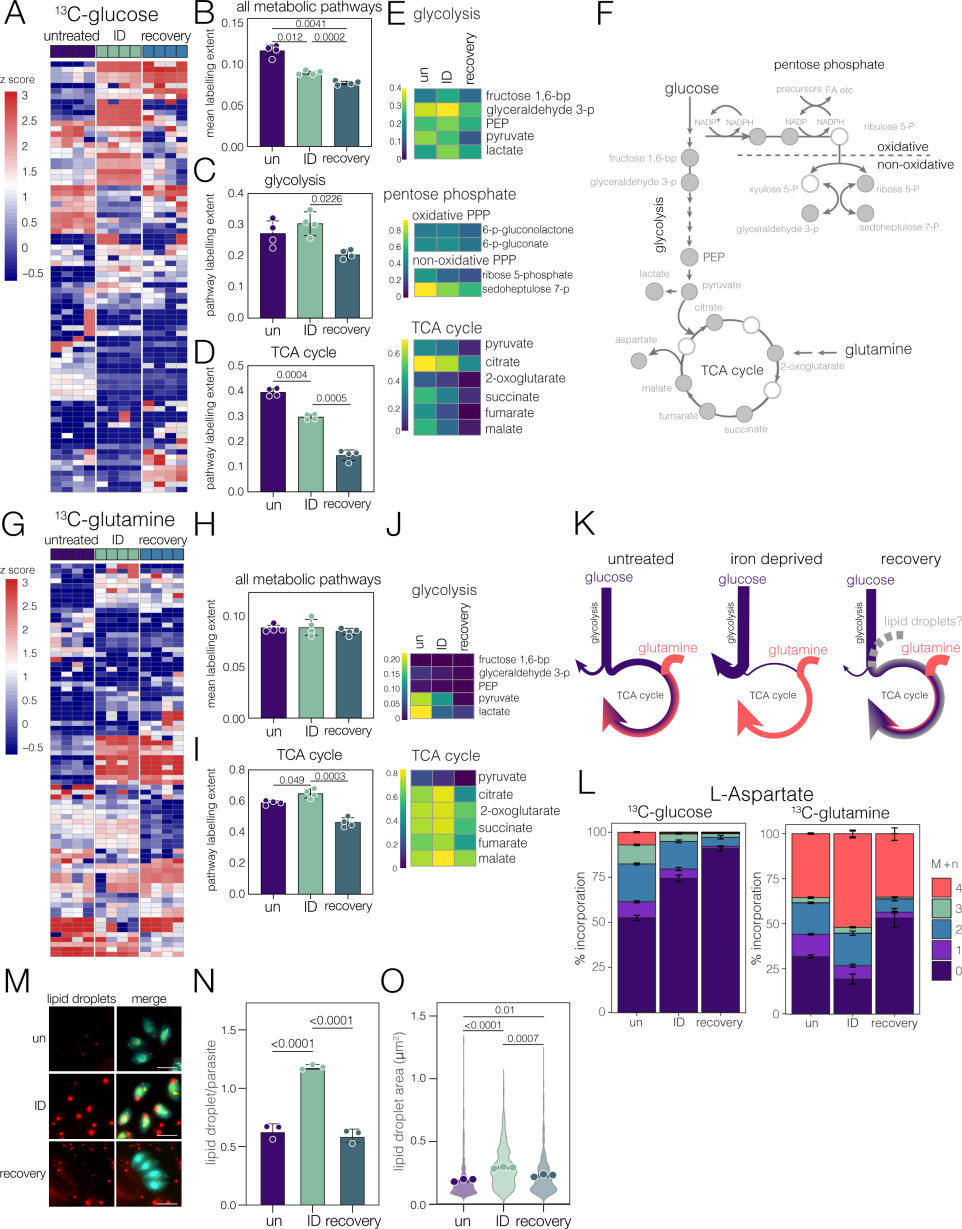
Stable isotope labeling reveals changes in the central carbon metabolism. (**A**) Clustered heatmap of z-score-transformed ^13^C glucose labeling extent (LE) for the top 80 most variable metabolites (by SD between conditions). Clustering was performed based on Euclidean distance at both the replicate and metabolite levels. Bar graphs of average glucose LE for all metabolites (**B**), glycolysis (**C**), and for TCA cycle (**D**) for each condition. Bar represents mean ± SD; Points represent replicates, and *P*-values were obtained from one-way Brown-Forsythe and Welch ANOVA with Dunnett’s correction. (**E**) Pathway heatmaps representing the average labeling extent across treatment conditions for glycolysis, pentose phosphate pathway, and TCA cycle. (**F**) Curated pathway schematic of central carbon metabolism showing key metabolites; white circles were not identified in our data set. (**G**) Clustered heatmap of z-score-transformed ^13^C glutamine LE for the top 80 most variable metabolites. Heatmaps were generated (**A**). Bar graphs of average glutamine LE for all metabolites (**H**) and the TCA cycle (**I**). Bars represent mean ± SD; points represent replicates. *P* values were obtained from one-way Brown-Forsythe and Welch ANOVA with Dunnett’s correction. (**J**) The pathway heatmaps for the average LE for glutamine in glycolysis and the TCA cycle. (**K**) Schematic of models of central carbon metabolism in control, iron-depleted, and recovery parasites. (**L**) Stacked bar graphs of aspartate isotopologue distributions from ^13^C-glucose (left) and ^13^C-glutamine (right) labeling. Bar colors represent the metabolite + number of labeled carbons (**N**) in each experimental condition. Bars represent mean ± SD. (**M**) Images of *Toxoplasma* (cyan, DNA is shown in yellow) with lipid droplets stained with Nile Red (red). Scale bar: 5 µm. Lipid droplet number (**N**) and area (**O**), which shows the area of all droplets (in violin), with replicate averages as points; bar represents mean ± SD. Results from three independent replicates. *P* values (from averages) from one-way ANOVA with Šidák correction.

To gain a more in-depth understanding of the metabolic changes in *Toxoplasma* upon iron depletion, we performed stable isotope labeling using ^13^C-labeled glucose or glutamine (Table S4), as previously described (67). Labeling of untreated, ID, or recovery parasites was reproducible as demonstrated by PCA analysis of total labeled carbon incorporation (Fig. S3D and E). Metabolite isotopologue abundances were normalized to the total metabolite abundance in each sample (Table S5), and those with significant changes in isotopologue distribution (adj. *P*-value < 0.01 from PERMANOVA) and greatest effect size (*R*^2^ > 0.65) (Fig. S3F and H) were identified, and changes in the top 80 (based on SD of z scores) metabolites were visualized by heatmaps (Fig. 5A and G). These highlight reproducible differences in carbon metabolism between untreated and iron-deprived parasites. To determine the changes in metabolic pathways, we performed pathway analysis, which revealed significant changes in carbon incorporation in the TCA cycle and pentose phosphate pathways (Fig. S3G and I) and in amino acid metabolism, as seen above in the quantitative metabolomics.

To determine how ^13^C-glucose was used by *Toxoplasma* in iron deprivation, we quantified average label incorporation, first across all detected metabolites, and found a significant (*P* = 0.012, one-way ANOVA, Dunnett’s correction) decrease in global incorporation upon iron deprivation (Fig. 5B). By examining individual pathways, we see no change in label incorporation in glycolysis (Fig. 5C), showing that flux through this pathway is maintained. However, we saw a significant (*P* = 0.0004, one-way ANOVA, Dunnett’s correction) decrease in labeled glucose in the TCA cycle (Fig. 5D). This suggests that although glycolysis proceeds, there is a disconnect in carbon flux between glycolysis and the TCA cycle.

Examination of individual metabolites revealed no major changes to the incorporation of glucose-derived carbon into glycolytic intermediates, including pyruvate (Fig. 5E and F). We do, however, see a significant increase in the incorporation of glucose-derived ^13^C into lactate in iron-depleted parasites (*P* = 0.0056, two-way ANOVA, Dunnett’s correction), suggesting a prioritization toward energy generation and away from carbon entering the TCA cycle through pyruvate. Labeling of the pentose phosphate pathway was incomplete; however, we obtained incorporation data for four canonical metabolites (Fig. 5E and F). We observed similar labeling of metabolites from the oxidative phase, but significantly depleted incorporation in the non-oxidative phase. The oxidative phase is critical for the regeneration of NADPH, which is then used in antioxidant defense and, importantly, in the reductive reactions of fatty acid synthesis (68, 69).

In contrast, labeling with ^13^C-glutamine revealed no difference in ^13^C incorporation across all pathways (Fig. 5H); however, in iron deprivation, we saw a small but significant (*P* = 0.049, one-way ANOVA, Dunnett’s correction) increase in label incorporation in the TCA cycle (Fig. 5I). Examination of the individual metabolites demonstrated almost no incorporation into glycolytic intermediates (Fig. 5J and K) under any conditions, demonstrating that there is little utilization of gluconeogenesis by *Toxoplasma.* However, in untreated parasites, glutamine is effectively incorporated into TCA cycle intermediates (Fig. 5J and K). Upon iron deprivation, this increases, suggesting that glutamine is making a larger contribution to the generation of these metabolites, and in combination with the results above, there is less dilution of the labeled carbon from unlabeled glucose upon iron depletion.

Taken together, we believe that these data represent a Warburg-like metabolic shift in which glycolysis and the TCA cycle are decoupled, and lactate dehydrogenase is deployed to regenerate NAD^+^ to sustain glycolysis, while aerobic respiration is inhibited (70). *Toxoplasma* possesses two lactate dehydrogenase isoforms (LDH1 and LDH2), of which LDH1 is expressed in tachyzoites (71). We see a strong induction of LDH1 abundance in iron depletion (Fig. S3J), supporting an acute metabolic adaptation to iron depletion

In addition to lactate formation through the Warburg effect, there is often a related increase in glutamine uptake and metabolism, which facilitates the formation of TCA cycle intermediates and macromolecular biosynthesis (72, 73). To determine how iron deprivation altered carbon incorporation into essential macromolecules, we examined L-aspartate, produced directly from the TCA cycle intermediate oxaloacetate (Fig. 5L). We found that aspartate demonstrated less incorporation from glucose, but more from glutamine, which supported our hypothesis of a disconnect between glycolysis and the TCA cycle.

### Recovery from iron depletion uses stored carbon

We also included parasites recovering from iron depletion after 24 h in our labeled metabolomics. Our expectation was that these parasites would be metabolically similar to control parasites, as their mitochondrial morphology had returned to normal. However, PCA analysis shows that these parasites form a distinct cluster from both our control and iron-depleted parasites (Fig. S3D and E). When interrogating the labeling extent across the detected metabolome, we determined that iron-depleted parasites incorporated less glucose-derived carbon compared to control parasites, but recovering parasites incorporated significantly less even than iron-depleted parasites (Fig. 5B). We also saw a significant decrease in glutamine-derived carbon in the TCA cycle (Fig. 5I and J).

The dramatic loss of incorporation by both glucose and glutamine by the recovery parasites begged the question of the source of the unlabeled carbon. Previous work (23) demonstrated that iron deprivation leads to the accumulation of lipid droplets in *Toxoplasma*. Lipid droplets can act as dynamic energy stores that can be liberated to support macromolecular biosynthesis (74–76), and given that our labeling began after these droplets had formed, they would consist of largely unlabeled carbon. To determine the fate of lipid storage upon recovery from starvation, we examined parasite lipid droplets. We confirmed previous results, which showed that iron starvation leads to a significant increase in lipid droplet number and area (*P* = <0.0001, one-way ANOVA, Šidák correction) (Fig. 5M, N, and O). Interestingly, at 24 h post-iron depletion, both the number and area of lipid droplets had fallen to the untreated levels. These data, together with the changes in label incorporation above, suggest a mobilization of carbon from these stores, which we hypothesize explains the significant decrease in macromolecule labeling in our recovery parasites.

### Glutamine restriction is protective under low iron.

**Fig 6:**
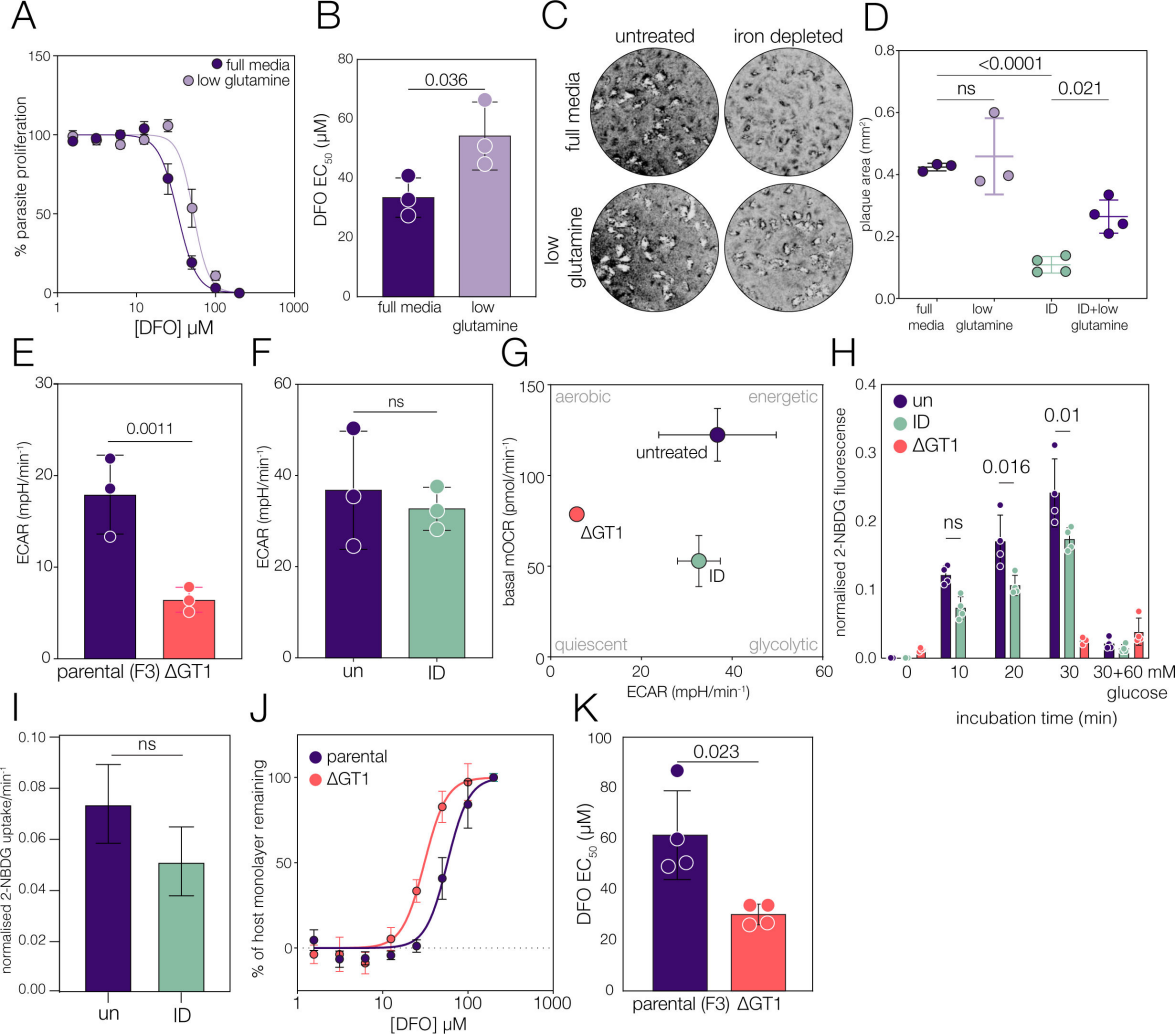
Glucose, but not glutamine, is required for survival under low iron. (**A**) Fluorescent growth assays to quantify sensitivity to DFO. Mean parasite proliferation in full media (dark) or low glutamine media (light), *n* = X, ±SEM. (**B**) Bar graph of DFO EC_50_, and each point represents a replicate; bars represent mean ± SD. *P*-value from a two-tailed paired *t*-test. (**C**) Representative plaque assays to assess the growth of ID parasites in different glutamine conditions. (**D**) Quantification of plaque area; bars represent mean ± SD, and *P* values were obtained from Brown-Forsythe and Welch ANOVA. No difference was observed between full media and low glutamine groups under iron-replete conditions (*n* = 3). Co-limitation of glutamine and iron significantly increased plaque area compared to ID alone (*n* = 4). (**E**) Extracellular acidification rate (ECAR) measured by Seahorse assay for parental and ΔGT1 parasites; bars represent the mean ± SD, and *P*-values are obtained from two-tailed unpaired *t*-test (*n* = 3). (**F**) ECAR was assessed in untreated and iron-depleted (ID) parasites, and no significant difference was detected using a two-tailed unpaired *t*-test (*n* = 3). (**G**) Metabolic map with points representing the mean basal mitochondrial oxygen consumption rate (mOCR) on the *y*-axis and mean ECAR on the *x*-axis, ± SD, *n* = 3. (**H**) Geometric mean parasite fluorescence upon incubation with fluorescent glucose analog 2-NBDG. Points indicate biological replicates; bars represent mean ± SD. *P* values were obtained from two-way ANOVA with Šídák correction. (**I**) Mean hill slope of 2-NBDG uptake, *n* = 4. (**J**) Quantification of monolayer disruption after infection upon DFO treatment. Points represent mean ± SEM, and data were fitted with a non-linear regression model (*n* = 4). (**K**) Mean DFO EC_50_ for parental and ΔGT1 parasites. Bars represent mean ± SD; *P* values were obtained from a two-tailed paired *t*-test (*n* = 4).

We found that iron deprivation leads to significant changes in parasite carbon metabolism; however, this cannot reveal the relative importance to parasite fitness of these pathways. To determine their significance to parasite survival and replication, we assessed how altering parasite carbon sources changed sensitivity to iron deprivation. Although *Toxoplasma* takes up glutamine from its environment (2)*,* the parasite can grow in the absence of glutamine (7). To determine the importance of glutamine uptake in low iron conditions, we tested the sensitivity of glutamine-restricted (0.4 mM glutamine, 10% of normal conditions) parasites to iron depletion. Restriction of glutamine led to a significant (extra-sum-of-squares *F* test, *P* <0.0001) protective effect for iron-deprived parasites, with significantly more DFO required to restrict parasite growth (IC_50_ diff = +20.75 µM) (Fig. 6A and B). We confirmed this result by plaque assay; as expected, limiting glutamine had no impact on parasite growth, while iron deprivation caused a significant decrease in plaque area (7) (Fig. 6C). However, glutamine restriction was able to partially rescue (*P* = 0.018, one-way ANOVA) plaque area upon iron deprivation (Fig. 6C and D), although there was no effect on plaque number (Fig. S5A), suggesting that glutamine restriction is protective for iron deprivation.

The metabolism of glutamine results in the production of ammonia as a by-product of the conversion to a-ketoglutarate (77). To investigate this further, we replaced glutamine with the synthetic dipeptide alanyl-glutamine (GlutaMAX), which breaks down more slowly, preventing ammonia buildup but providing increased alanine to the cells. In this case, changes in alanyl-glutamine concentration did not sensitize cells to iron restriction (Fig. S5B). The difference between glutamine and GlutaMAX supports our hypothesis that carbon source availability has a direct impact on sensitivity to iron restriction.

These data show that glutaminolysis is dispensable to iron-starved parasites and suggest that excess glutamine catabolism in iron-deprived cells has a detrimental effect on growth.

### *Toxoplasma* requires glucose metabolism during iron deprivation

Unlike mitochondrial respiration, energy generation through glycolysis—although less efficient—does not require iron. To assess if iron-deprived parasites were utilizing glycolysis, we quantified the extracellular acidification rate (ECAR) using the Seahorse analyzer. This gives an indirect assessment of glycolysis based on media acidification after lactic acid excretion and can be used to assess shifts in glucose metabolism (53). To confirm that ECAR is an effective proxy for glycolysis, we generated a parasite line that lacks the major glucose transporter, GT1 (TGGT1_214320) (Fig. S5C), which is expected to be significantly inhibited in glucose uptake and consequently has a very low rate of glycolysis (6, 7). ΔGT1 parasites had a small (unpaired *t-*test, *P* = 0.031) defect in basal oxygen consumption rate (Fig. S5D and E) and had a functional mitochondrial electron chain, as demonstrated by no changes in complex IV activity (Fig. S5F) or ATP synthase complex integrity (Fig. S5G). However, loss of GT1 led to a significant drop (unpaired *t-*test, *P* = 0.037) in extracellular acidification rate (Fig. 6E), validating ECAR as a proxy for glycolysis in *Toxoplasma*. In contrast, although iron-deprived parasites had a significant defect in mitochondrial respiration, extracellular acidification was maintained (Fig. 6F), pushing cells toward a more glycolytic phenotype, as could be seen from the metabolic map (Fig. 6G).

Glycolysis requires glucose; to quantify glucose uptake, we made use of the fluorescent glucose analog 2-NBDG (78). To validate this approach, we confirmed that ΔGT1 parasites were unable to take up 2-NBDG and that 2-NBDG uptake could be entirely blocked by excess (60 mM) unlabeled glucose (Fig. 6H). Both untreated and iron-deprived parasites took up 2-NBDG, although we saw significantly (*P* < 0.05, two-way ANOVA with Šidák correction) less signal at both 20 and 30 min post-incubation in iron-deprived parasites (Fig. 6H). It is possible that this is linked to decreased extracellular viability of ID parasites; however, calculating the rate of 2-NBDG uptake showed no significant change in 2-NBDG uptake between conditions (Fig. 6I ; Fig. S5H), demonstrating that iron-deprived parasites can effectively take up glucose.

In *Toxoplasma*, although the glycolysis pathway has been maintained, it is typically dispensable under standard growth conditions (6). To determine if glycolysis becomes more fitness-conferring under low iron, we tested the sensitivity of the parasite to iron chelation under media containing high (4.5 mM) or low (1 mM) glucose. We saw a small but significant (*P* = 0.0272, two-tailed *t*-test) increase in sensitivity to iron chelation in low glucose media (Fig. S5I). However, changing extracellular glucose concentrations may not directly impact the parasite so we made use of the ΔGT1 parasite line. We find loss of the glucose transporter makes the parasites significantly (*P* <0.0001, extra-sum-of-squares F test) more sensitive to iron chelation (IC_50_ diff. = −31.24 µM) than the parental parasite line (Fig. 6J and K), demonstrating that glycolysis is required for replication under low iron conditions.

These results demonstrate the importance of central carbon metabolism in parasite replication under low iron, and highlight the connections between metabolism and iron utilization in this parasite.

## DISCUSSION

Here, we have mapped the effects of acute iron deprivation on the proteome and metabolism of *T. gondii.* We find a significant remodeling of the parasite proteome, which is poorly correlated with changes in the transcriptome. Poor correlation between protein and transcript levels has previously been observed in other eukaryotes (38, 39), especially upon stress when changes in mRNA availability and translation can lead to rapid changes in the proteome (79).

One potential mechanism for this discordance is the rapid and profound drop in parasite translation upon iron deprivation. Translational inhibition upon iron deprivation has been observed in other organisms (11, 80, 81); however, the mechanisms and pathways vary. In mammalian cells, translation is inhibited by iron deprivation via signaling through the mTOR pathway (11), which is absent from *Toxoplasma*. In yeast, iron-mediated translation inhibition is initiated by repression of Rli1 (an ABCE1 homolog) through RNA-binding proteins (81). In *Toxoplasma*, we find that ABCE1 is significantly downregulated upon iron depletion; although this decrease in abundance is seen after translational repression. However, ABCE1 can be regulated beyond abundance (82, 83), and here we did not quantify activity directly. While the temporal regulation of ABCE1 and translation suggests a link, due to the involvement of numerous iron-containing proteins in regulating translation, we cannot define the mechanism linking iron availability and translation. In mammalian cells, the integrated stress response (ISR) has an important role in the response to low iron (84), and a number of previous studies have found evidence for an equivalent ISR in *Toxoplasma* (85–88), making the ISR an attractive candidate for mediating iron-dependent translational inhibition. Future work will seek to integrate how the parasite senses iron and how translation is regulated in response.

A key destination for iron within the parasite is the mitochondrion, where iron is required in enzymes of the TCA cycle and the ETC (24, 61, 89*).* In *Toxoplasma*, iron deprivation leads to dynamic and reversible changes in parasite mitochondrial morphology, membrane potential, and in respiration. While crucial for oxidative phosphorylation, mitochondrial polarization is also vital for the import of proteins, ions, and metabolites (90, 91), and depolarization may be linked to the metabolic phenotypes observed in this study. We also see a drop in both basal and maximal oxygen consumption, which is also seen upon depletion of the major iron and zinc importer ZFT (33), highlighting the importance of iron to mitochondrial respiration.

We see significant changes in metabolism, including the accumulation of citrate and fumarate. Citrate is metabolized by aconitase, which we have previously shown has reduced activity in iron depletion (24), and here, we show that iron-depleted parasites are more sensitive to aconitase inhibition, suggesting a mechanism for its accumulation. Citrate also plays a key role in the cytosol, where it is used to generate acetyl-CoA through the action of ATP-citrate lyase (ACL), an important step in fatty acid metabolism (92, 93). Citrate accumulation has previously been associated with an increase in lipid droplets in iron-depleted mammalian cells (59, 94), which we also see here. ACL is downregulated in iron deprivation, which could also contribute to an additional accumulation of cytosolic citrate. Alongside downregulation of ACL, we see upregulation of acetyl-CoA synthase (ACS), which provides an alternate supply of cytosolic acetyl-CoA from host-derived acetate to supply fatty acid synthesis (30). ACS and ACL form a synthetically lethal pair in *Toxoplasma* (92); therefore, their divergent regulation at the protein level suggests that scavenged acetate is the major driver of lipid droplet accumulation in iron-deprived parasites (23), at the cost of citrate accumulation. These lipid droplets may function as an important energy store during this acute nutrient deprivation, as they are apparently mobilized upon nutrient replacement.

We believe that these lipid droplets then become important as parasites recover from acute iron deprivation. Stable isotope labeling of recovering parasites showed a significant decrease in total glucose incorporation, particularly into the TCA cycle. These recovering parasites have normal mitochondrial morphology and reinitiate replication; hence, we believe that this represents the incorporation of carbon from an unlabeled source. We suggest that recovering parasites represent the incorporation of carbon from an unlabeled source. We suggest that recovering parasites mobilize stored carbon from lipid droplets for incorporation into cellular macromolecules, which coincides with lipid droplet depletion. Lipid droplets can also perform functions beyond acting as versatile energy stores, including managing cellular stress and acting as protein stores (74, 95, 96). It is therefore possible that lipid droplets play an important role in parasite survival in iron starvation, beyond serving as an energy store; however, future studies will examine the processes required to mobilize this carbon store.

*Toxoplasma* balances glycolysis and glutamine catabolism to generate energy for invasion and replication (2, 7). Previously, it has been reported that extracellular parasites use accumulated GABA as an additional carbon source to support motility (2). We unfortunately did not identify GABA in our metabolomics datasets; hence, we cannot assess if GABA plays a role in metabolism in iron-depleted parasites. Our stable isotope labeling revealed a significant increase in the incorporation of glucose-derived carbon into lactate with a corresponding decrease in the incorporation into the TCA cycle. Additionally, our labeling of iron-depleted parasites revealed a likely compensatory increase in glutamine-derived carbon incorporated into the TCA cycle and aspartate synthesis. This represents a Warburg-like shift in iron-depleted metabolism, whereby lactate production is used to regenerate NAD+ that sustains glycolysis as the primary energy-generating process, and glutamine is deployed in anaplerosis in the TCA cycle.

Despite the metabolic shift, we observed profound differences in the importance of these carbon sources to iron-depleted parasites. *Toxoplasma* with restricted glucose uptake (ΔGT1) are significantly more susceptible to iron depletion, while glutamine restriction, which is predicted to increase flux through glycolysis (7, 97), is protective against low iron. Sustained glutaminolysis while parasites are repressing translation and DNA replication may lead to the accumulation of potentially toxic metabolites, such as ammonia, which may inhibit parasite growth. Recently, it was shown that genetic manipulation of glycolysis inhibited virulence in a mouse model, although growth *in vitro* was not affected (98). Iron is frequently withheld during infection (99), and potentially limited iron during *in vivo* infection could help explain some of the disparate phenotypes seen. This highlights the importance of metabolic flexibility to *Toxoplasma*, providing a hypothesis for the parasite’s maintenance of these overlapping and frequently redundant pathways.

Here, we show for the first time the consequences of acute iron deprivation on *Toxoplasma*. Parasites rapidly inhibit translation, leading to proteomic remodeling and a metabolic switch. The battle for iron is an essential aspect of pathogenesis and survival in iron-limited environments important for a range of pathogens, including *Toxoplasma*.

## MATERIALS AND METHODS

### *T. gondii* and host cell maintenance

*T. gondii* tachyzoites were maintained at 37°C with 5% CO_2_ and were grown in human foreskin fibroblasts (HFFs) cultured in high-glucose (4,500 mg/L) Dulbecco’s modified Eagle’s medium (DMEM), supplemented with 3% (D3) heat-inactivated fetal bovine serum (FBS), 4 mM L-glutamine, 100 units/mL penicillin, and 100 µg/mL streptomycin. HFFs were passaged in DMEM with 10% FBS (D10), 4 mM glutamine, and penicillin/streptomycin. For iron deprivation (ID), confluent host cells were incubated with D3 media containing 100 µM deferoxamine (Deferoxamine mesylate salt, Sigma) for 24 h prior to infection. Iron-complemented host cells (ID+FAC) were incubated with both 100 µM deferoxamine and 200 µM ferric ammonium citrate (FAC). For low glutamine conditions, D3 media was supplemented with 0.4 mM L-glutamine.

### Strain construction

To generate the *gt1* knockout strain, a gRNA targeting the N-terminus of TGGT1_214320 was identified using ChopChop (https://chopchop.cbu.uib.no/) and cloned into the Tub-Cas9YFP-pU6-ccdB-tracrRNA plasmid (100) via BSaI restriction site using primers P1 and P2. (sgRNA sequences are listed in Table 2).

**TABLE 2:** List of gRNA and primers used in this study

Number	Description	Sequence (5′–3′)
P1	gRNA seq for GT1 FW	aagttTTCGAAGGACGCGTTCATCAg
P2	gRNA seq for GT1 RV	aaaacTGATGAACGCGTCCTTCGAAa
P3	To amplify DHFR with overhangs for GT1 FW	CAGTATACCCGTCTTCGAGACCCCGACTTGTTTCGAAGGACGCGTTCATCaagcttcgccaggctgtaaatcc
P4	To amplify DHFR with overhangs for GT1 RV	ACACGAAAAAAAACTGAAATGGGCGCGACGACAGACAAAATGTCGCTCAAggatcgatcccccggtttgc
P5	KO test (GT1 5’UTR) FW	ATCGTTCTTTCCGAGAGACG
P6	KO test (DHFR ORF) RV	CACGGTTATCAAACCCGAG

The repair template containing DHFR was amplified by PCR with primers P3 and P4 (Table 2) containing overhangs encoding 50 bp of homology for the regions upstream and downstream of the TGGT1_214320 ORF. The ΔGT1 strain was generated in a TATi/ΔKu80 background (101) by transfecting with 50 µg of gRNA plasmid generated above and the PCR-amplified repair template containing DHFR ORF. Transfections were carried out using a Gene Pulse BioRad electroporator. Parasites were selected using 2 µM pyrimethamine and cloned by serial dilution. Positive clones were tested by PCR using primers P5 and P6 (Table 2).

### Immunoblotting

For western blot analysis, parasites were grown on untreated, iron-deprived, or iron-complemented host cells for 24 h. Parasites were mechanically released by scraping and passing 2–3× through a 25 g needle, filtered through a 3-µm polycarbonate filter to remove host cell debris, and washed. For puromycin incorporation, 5 × 10^6^ parasites were incubated in media containing 10 µg/mL puromycin (P8833, Sigma) for 30 min at 37°C prior to lysate collection. Whole cell lysates were prepared by incubating in lysis buffer for 30 min on ice (150 mM sodium chloride, 1% Triton X-100, 0.5% sodium deoxycholate, 0.1% sodium dodecyl sulfate (SDS), and 50 mM Tris, pH 8.0). An appropriate volume of 4× SDS loading dye was added, and samples were boiled for 10 min. Proteins were separated on a 10% SDS gel before being transferred to the nitrocellulose membrane for 1 h, blocking for 1 h at room temperature in blocking buffer (4% milk in PBST). Membranes were incubated overnight with anti-puromycin (1:1,000, MABE343, Merck), anti-*Tg*CDPK1 (1:10,000 [102]), anti-HA (1:1,000, 11867423001, Merck), and anti-Flag (1:1,000, MA191878, Invitrogen) antibodies in blocking buffer and washed three times in PBST. Fluorescent secondary antibodies (IRDye800 and IRDye680, LI-COR) were used to visualize proteins using the Odyssey CLx. For puromycin incorporation, total protein was visualized using ponceau, prior to probing, and quantified by densitometry analysis using ImageJ (Fiji) and used to normalize puromycin signal. Quantification for proteins of interest was normalized to respective loading controls, and each condition was plotted relative to the sum of normalized protein quantified in each replicate to show variation in controls between replicates, as previously described (103).

Complex-V (ATP-Synthase) assembly was detected using Blue-Native PAGE followed by western blot. Briefly, freshly extracellular parasites were solubilized in 1% β-DDM (wt/vol) in 750 mM aminocaproic acid solution on ice for 10 min. The samples were centrifuged at 16,000 × *g* at 4°C for 30 min in a bench-top centrifuge. The resulting supernatants were combined with Coomassie G250 to a final concentration of 0.25%, and proteins were resolved on pre-cast 4%–16% NativePAGE Bis-Tris gels (Invitrogen). The gel was run at 80 V for 1 h, followed by 250 V for 2 h. NativeMark Unstained Protein Standard (Invitrogen) was used as a molecular weight marker. Proteins were transferred to a 0.45 μm PVDF membrane (Hybond, Merck) using the wet transfer method at 100 V for 1 h in Towbin transfer buffer (25 mM Tris, 192 mM glycine, 10% methanol). Membranes were blocked using 5% (wt/vol) skimmed milk in PBS with 0.1% Tween-20, probed with a primary rabbit anti-ATP-β antibody (1:2,000, Agrisera AS05085), followed by a horseradish peroxidase (HRP)-conjugated secondary anti-rabbit antibody (1:10,000, Promega W4011). Detection was done using the Pierce ECL substrate (Thermo Scientific).

### OPP incorporation

ΔKu80 parasites were grown on coverslips with untreated, iron-deprived, or iron-complemented host cells for 24 h. Cells were then labeled with 20 µM Click-iT O-propargyl-puromycin conjugated to Alexa Fluor 488 (OPP, Invitrogen) for 30 min at 37°C. Cyclohexamide (CHX) was used as a negative control, with parasites incubated with 100 µM CHX for 20 min before OPP labeling. Coverslips were washed with PBS before being fixed with 4% formaldehyde and permeabilized with 0.5% Triton X-100. Using a click chemistry reaction, OPP was labeled following the manufacturer’s instructions with Alexa Fluor picolyl azide for 30 min at room temperature, protected from light. Coverslips were mounted with Fluoromount with DAPI (Southern Biotech) and imaged using a Zeiss LSM 880 inverted confocal microscope and Zen black software (Zeiss). Images were processed and analyzed with an automated macro with ImageJ (Fiji).

For the OPP incorporation and ABCE1 time course experiment, host cells on coverslips were left untreated or pretreated for 24 h prior to infection with ABCE1-HA parasites. After 24 h, the iron-depleted medium was removed, and wells were washed with fresh media twice before fresh media were added to begin the recovery process. At 6, 12, 18, and 24 h after infection or recovery, coverslips were labeled with OPP as described in the previous paragraph. Coverslips were then incubated in 0.5% BSA in PBS containing anti-HA (1:1,000, Merck 11867423001) and anti-TgIMC1/TgSAG1/TgCDPK1 (1:1,000) antibodies. Coverslips were then washed with PBS before incubation with anti-rat and anti-mouse secondary antibodies conjugated to Alexa Fluor 594 or Alexa Fluor 647, respectively (both 1:1,000). After further PBS washes, cells were then mounted onto slides with Fluoromount with DAPI (Southern Biotech). Images were obtained using the Zeiss LSM 880 inverted confocal microscope using Zen black software (Zeiss). Images were obtained from four replicates, with each replicate consisting of the vacuoles contained within five images. OPP incorporation and HA abundance were quantified in parasite vacuoles using automated macros within ImageJ (Fiji). For each image, a parasite mask was generated by thresholding on CDPK1 (a cytosolic marker). The mask generated by cytoplasmic CDPK1 was then used to quantify the mean fluorescence intensity in the 488 channel in which OPP-Alexa Fluor 488 was imaged. For HA abundance, the HA signal contained within host cell nuclei was used to background-adjust the signal obtained from the parasite within the same image. All macros available upon request.

### Seahorse extracellular flux analysis

Mitochondrial oxygen consumption rate (mOCR) and extracellular acidification rate (ECAR) were measured with a Seahorse XF HS Mini analyzer (Agilent) as previously described (49, 53). Briefly, parasites were grown as indicated for 24 h before being released and filtered as above and washed with Seahorse XF DMEM base medium supplemented with 5 mM glucose and 1 mM glutamine. A total of 1.5 × 10^6^ parasites were added to each well of an eight-well XF miniplate pretreated with poly-L-lysine, and OCR and ECAR were quantified. The background, non-mitochondrial oxygen consumption rate was defined as that present after the addition of 1 µM atovaquone. Basal and maximal mOCR were calculated by taking the OCR measurement before (basal) or after (maximal) the addition of 1 μM FCCP and subtracting the background rate. Each experiment was performed in triplicate, with three biological repeats.

### Fluorescence growth assays

HFF cells were seeded onto clear-bottom black 96-well plates (Thermo Scientific), allowed to reach confluency, and infected with 1,000 mNeon or tdTomato (32) parasites per well. After 2 h at 37°C, the media were replaced with the indicated concentrations of the drug added. After 4 days of infection, parasite fluorescence was measured using a PHERAstar FS microplate reader (BMG LabTech) at 488 or 594 nm emission for mNeon and tdTomato, respectively. Experiments were performed in technical triplicate with at least three biological replicates. Uninfected wells served as blanks to remove background fluorescence, and values were normalized to infected, untreated wells of the appropriate condition to enable comparisons between lines and conditions. Dose-response curves and EC_50_ calculations were performed in GraphPad Prism 10, using the inhibitor vs. normalized response (variable slope) model. Extra sum of squares F-tests or *t*-tests were performed to test the differences in best-fit IC_50_ values between groups.

### Flow cytometry analysis

Glucose uptake was assessed by flow cytometry using the fluorescent glucose analog 2-NBDG (Invitrogen, N13195). ΔKu80::tdTomato or ΔGT1 parasites were grown on untreated or iron-depleted host cells for 24 h. Parasites were released by scraping and syringing infected host cells, and parasites were filtered from host material through a 3-µm filter. Parasites were washed in glucose-free media before being resuspended in DMEM supplemented with 2.5 µM l-glutamine only. As a competitive inhibition control, parasites were resuspended in 2.5 µM l-glutamine and 60 mM glucose. Parasites were then left unstained or were incubated with 1 mM 2-NBDG for 10, 20, or 30 min at 37°C. Competition controls were incubated with 1 mM 2-NBDG for 30 min. Parasites were analyzed on a BD FACSCelesta Flow Cytometer, and data were acquired using FACSDiva software (BD Biosciences). Parasites were gated on forward and side scatter and on red fluorescence (only for tdTomato parasites). All data were analyzed using FlowJo v10 (BD Biosciences), and the geometric mean of the BB515 channel was used to quantify 2-NBDG uptake. Each data point was normalized to the sum of its replicate to both show variation among replicates while also controlling for variation in total fluorescence between them (103).

### Immunofluorescence assay

Immunofluorescence assays were performed on both intracellular and egressed extracellular tachyzoites. Intracellular *T. gondii* parasites were grown on coverslips pre-seeded with HFFs before being fixed with 4% paraformaldehyde for 10 min at room temperature. Cells were permeabilized for 10 min in 0.5% Triton X-100 in PBS before being stored in 2% BSA, 0.2% Triton X-100 at 4°C overnight. Coverslips were then incubated with primary antibodies in PBS; rat anti-HA (1:1,000, Merck 11867423001), guinea pig anti-*Tg*CDPK1 (1:5,000), mouse anti-*Tg*IMC1/*Tg*SAG1 (1:1,000), rabbit anti-*Tg*TOM40 (1:1,000), or Nile Red (0.5 μg/mL, 10464311, ThermoScientific). Coverslips were then washed with PBS before incubation with secondary antibodies raised against the host of the corresponding primary antibody, conjugated to Alexa Fluor 488, Alexa Fluor 594, or Alexa Fluor 647 (all 1:1,000). After further PBS washes, cells were mounted onto slides with Fluoromount with DAPI (Southern Biotech). Images were obtained using the Zeiss LSM 880 inverted confocal microscope using Zen black software (Zeiss) or with a Leica DiM8 (Leica Microsystems) microscope and processed using LasX (Leica Microsystems). Images were processed and analyzed with automated macros in ImageJ (Fiji).

For time course analysis of mitochondrial morphology, host cells on coverslips were left untreated or pretreated for 24 h prior to infection with ΔKu80 parasites. After 24 h, the iron-depleted medium was removed from the recovery wells, and the cells were washed with fresh media twice before fresh media were added to begin the recovery process. At 6, 12, 18, and 24 h after infection or recovery, the cells were fixed and permeabilized as described in the previous paragraph. Coverslips were stained with anti-TOM40 (1:1,000) and anti-CDPK1 (1:5,000) antibodies before washing in PBS before incubation with secondary antibodies.

### MitoTracker imaging

Parasites treated as above were mechanically released, washed once with PBS, and incubated in the presence of 10 µM Hoechst 33342 (ThermoFisher Scientific, H3570) and 50 nM MitoTracker Deep Red (Invitrogen, M22426) for 10 min at 37°C on poly-L-lysine-treated glass bottom dishes. For imaging of host mitochondrial association, ΔKu80:tdTomato parasites were cultured for 24 h on untreated, ID, or ID+FAC host cells before incubation with 50 µM MitoTracker Green FM for 15 min at 37°C. Parasites were imaged live using a Leica DiM8 (Leica Microsystems) microscope and processed using LasX (Leica Microsystems). Intensity was analyzed manually using ImageJ (Fiji).

### Crystal violet growth assays

HFF cells were seeded onto clear 96-well plates (Thermo Scientific), allowed to reach confluency, and infected with 1,000 ΔGT1 or parental TATi/ΔKu80 parasites per well. After 2 h at 37°C, the media were replaced with indicated concentrations of the drug added. After 4 days of infection, the media were removed, and the cells were fixed in 20 µL ice-cold methanol before being stained for at least 2 h in 0.5% crystal violet in 20% ethanol solution. Wells were then washed with sterile water before being left to dry. Absorbance was measured in a PHERAstar FS microplate reader (BMG LabTech). Experiments were performed with six technical repeats with four biological replicates. Unstained wells served as blanks to remove the background, and values were normalized to infected wells treated at 200 µM DFO. Dose-response curves and IC_50_ calculations were performed in GraphPad Prism 10, using the inhibitor vs. normalized response (variable slope) model. Extra sum of squares F-tests were performed to test differences in best-fit IC_50_ values between groups and differences in average IC_50_ were analyzed by *t*-test.

### Plaque assays

In total, 1,000, 500, or 250 parasites were dispensed onto confluent monolayers of HFF cells in six-well plates. Parasites were allowed to replicate for 5–7 days undisturbed before being washed with PBS, followed by fixation with ice-cold methanol for 10 min. Cells were stained using 0.5% crystal violet in 20% ethanol solution for at least 2 h at room temperature before washing with distilled H_2_O. Plates were imaged, and plaque numbers and sizes were quantified using ImageJ (Fiji).

### Proteomics sample preparation

To prepare samples for proteomics, 1 × 15 cm dishes for control conditions and 2 × 15 cm dishes for iron-deprived conditions were pooled to generate each replicate. For iron deprivation, confluent host cells in 15 cm dishes were incubated with D3 media containing 100 µM deferoxamine (deferoxamine mesylate salt, Sigma) for 24 h prior to infection. After 24 h of infection, the cells were washed twice in PBS to remove extracellular parasites, before being scraped and passed through a 25-guage needle to mechanically release the parasites. Host cell material was filtered from parasites by passing through a 3-µm filter. Parasites were collected by centrifugation at 3,000 × *g* for 25 min. Parasites were counted, and 4 × 10^7^ parasites were resuspended in lysis buffer (50 mM K phosphate buffer, 1% SDS, 1 mM EDTA, 1 mM DTT, 1× Universal nuclease [Thermo Scientific, 12156490], 1× Halt protease inhibitors (Thermo Scientific, 10720825) and left to incubate on ice for 30 min. Lysates were freeze-thawed three times in a dry-ice and ethanol bath to enhance lysis.

Insoluble material was removed from lysates by centrifuging at 20,000 × *g* at 4°C for 20 min and collecting the supernatant. Samples were then reduced in 25 mM TCEP (Fisher) for 10 min at 37°C and then alkylated with 25 mM iodoacetamide (Fisher) for 1 h at RT in darkness. Proteins were then precipitated by adding 12% volume ice-cold trichloroacetic acid (TCA) (Sigma) and incubated at −20°C overnight. Precipitated proteins were pelleted at 16,000 × *g* at 4°C and washed with agitation three times with ice-cold acetone. Samples were then resuspended in 100 mM triethylammonium bicarbonate (TEAB) (Fisher) and were then digested by a trypsin/Lys-C mix (Promega) at a 1:25 (wt/wt) ratio overnight at 37°C. Peptides were dried using a SpeedVac SPD1030 vacuum concentrator. Peptide samples were resuspended in 1% formic acid (#85178, Thermo Scientific) before being subjected to quality control.

### Mass spectrometry

Analysis of the peptides was performed on a Q Exactive HF Quadripole Orbitrap Mass Spectrometer (Thermo Scientific) coupled to a Dionex Ultimate 3000 RS HPLC system (Thermo Scientific). The buffers used were buffer A (0.1% formic acid in Milli-Q water [vol/vol]) and buffer B (80% acetonitrile [#83640.290], VWR), and 0.1% formic acid in Milli-Q water (vol/vol). An equivalent of 1.25 µg of peptides from each sample was loaded at 10 µL/min onto an Acclaim PepMap 100 C18 column (#164750, Thermo Scientific). This trapping column was washed for 6 min at the same flow rate with 1% buffer B and then switched in-line with a µPAC Neo column (#COL-NANO110NEOB, Thermo Scientific). Buffer B was set to 3.8% and then increased over the next 117 min to 41.3%; increased to 61.3%, 99% in 13-min and 10-min steps; the column was then washed for 17 min. Two blanks were run between each sample to reduce carryover. The column was kept at a constant temperature of 50°C.

The Q-Exactive HF was operated in positive ionization mode using an easy spray source. The source voltage was set at 1.90 kV, and the capillary temperature was 250°C. Data were acquired in data-independent acquisition mode as previously described (Doellinger et al., 2020), with little modification. A scan cycle comprised a full MS scan (345–1,155 *m*/*z*), resolution was set to 60,000, AGC target 3 × 10^6^, and maximum injection time 200 ms. MS survey scans were followed by DIA scans of dynamic window widths with an overlap of 0.5 Th. DIA spectra were recorded at a resolution of 30,000 at 200 *m*/*z*, using an automatic gain control target of 3 × 10^6^, a maximum injection time of 55 ms, and a first fixed mass of 200 *m*/*z*. Normalized collision energy was set to 25% with a default charge state set at 3. Data for both MS scan and MS/MS DIA scan events were acquired in profile mode.

### Proteomics data analysis

Analysis of the DIA data was carried out using Spectronaut (version 17.4.230317.55965, Biognosys, AG). The directDIA workflow, using the default settings (BGS Factory Settings) with the following modifications was used: decoy generation set to inverse, Protein LFQ Method was set to QUANT 2.0 (SN Standard), and Precursor Filtering was set to Identified (Qvalue); Precursor Qvalue Cutoff and Protein Qvalue Cutoff (Experimental) were set to 0.01; Precursor PEP Cutoff was set to 0.1, Protein Qvalue Cutoff (Run) was set to 0.05, and Protein PEP Cutoff was set to 0.75.

For the Pulsar search, settings were a maximum of two missed trypsin cleavages; PSM, Protein, and Peptide FDR levels set to 0.01; scanning ranges set to 300–1,800 *m*/*z* and Relative Intensity (Minimum) set to 5%; cysteine carbamidomethylation set as fixed modification and acetyl (N-term), deamidation (asparagine, glutamine), dioxidation (methionine, tryptophan), glutamine to pyro-Glu, and oxidation of methionine set as variable modifications. The databases used were *T. gondii* GT1 (reviewed, downloaded from ToxoDB on 14 September 2023).

Differential expression analysis was performed in RStudio using the ProteoDA package (104). Protein intensity values were evaluated using the proteiNorm tool (105) before being transformed using variance stabilizing normalization. Statistical analysis was performed using the limma package (106) in R. Proteins with an FDR adjusted *P*-value <0.05 and a fold change >1.5 (log_2_0.65) were considered differentially expressed. DISCO scores were calculated by integrating these proteomic data with transcriptomic data previously reported in a previous study (24). DISCO scores were computed as previously described (37) using the formula DISCO score = log_2_FCRNA × log_2_FCPROT × (log_10_*P*-adjRNA + log_10_*P*-adjPROT) × sign(log_2_FCRNA) × sign(log_2_FCPROT).

### Metabolomics sample preparation

For untargeted metabolite extraction, 2 × 15 cm dishes for control conditions and 4 × 15 cm dishes for iron-deprived conditions were pooled to generate each replicate. For iron deprivation, confluent host cells in 15 cm dishes were incubated with D3 media containing 100 µM deferoxamine (deferoxamine mesylate salt, Sigma) for 24 h prior to infection. After 24 h of infection, dishes were harvested.

For stable isotope labeling, 2 × 15 cm dishes for control conditions and 4 × 15 cm dishes for iron-deprived or recovery conditions were pooled to generate each replicate. Iron deprivation was achieved as described previously, while recovery was achieved by replacing media on iron-depleted cells with iron-replete media. After either 24 h of infection or recovery, cells were washed with PBS before being incubated at 37°C for 4 h with labeling media containing either 5.55 mM C^13^ glucose or 4 mM C^13^ glutamine.

To harvest all metabolomic samples, dishes were moved to a shallow container of ice water, and the media were rapidly replaced with ice-cold PBS twice to quench metabolism and remove extracellular parasites and old media. Cells were then scraped and passed through a 25-guage needle to mechanically release parasites. Parasites were filtered from host material through a 3-µm filter before centrifugation at 1°C for 25 min at 3,000 × *g*. Pellets were resuspended and washed in 1 mL ice-cold PBS before being counted using a hemocytometer; 4 x 10^7^ parasites were resuspended in 200 µL of a chloroform/methanol/water mixture (1:3:1 ratio). Samples were shaken for 1 h at 4°C and centrifuged at 20,000 × *g* for 5 min at 4°C. Metabolites in the supernatant were then collected.

### Metabolomics data analysis

Metabolites were quantified by LC-MS as previously described (107). In brief, samples were separated by high-performance liquid chromatography on a Dionex UltiMate 3000 RSLC system (Thermo) using a ZIC-pHILIC column (Merck). Mass spectrometry was performed using an Orbitrap Q Exactive (Thermo) with analysis performed in both positive and negative ionization modes. To improve metabolite identification, samples were run alongside internal standard mixtures that included metabolites from a wide range of metabolic pathways. Additionally, quality control pooled samples were also subject to fragmentation to acquire MS2 spectra using data-dependent acquisition (DDA).

Features were annotated using the mzMatch package in R along with the IDEOM software (108, 109). Features with a root squared deviation (RSD) greater than 40% in QC samples were removed, along with those with retention times < 3 min. Metabolites were annotated according to the Metabolite Standards Initiative (110) with level 2 (mass only) and level 1 annotations included in downstream pathway analysis. MSI level 1 annotations correspond to metabolites present in the mixture of 240 standards run alongside experimental samples. Peaks were also subjected to manual filtering to remove poor-quality peaks. Statistical analysis of untargeted data wa performed using the MetaboanalystR 4.0 package within RStudio (111). Features identified in only one condition have missing values imputed with 1/5 the minimum value detected. Median normalization, log_10_ transformation, and mean centering were performed on peak intensities from identified metabolites prior to hierarchical clustering and differential abundance analysis within MetaboanalystR 4.0. Pathway analysis was performed with MetaboAnalyst 6.0 using global test enrichment against the *T. gondii* KEGG pathways (112). Relative-betweenness centrality was used as a topology measure of pathway impact. Metabolites closer to each other in the pathway are assigned a higher pathway impact score (between 0 and 1).

Stable isotope labeling analysis was performed in RStudio. Isotopologue intensities were corrected for the natural abundance of isotopes using the AccuCor package in R (113). Isotopologue intensities were then normalized to the proportion of total metabolite intensity. To test for changes in isotopologue distributions, data were first centered log-ratio (CLR) transformed to mitigate for dependencies in the normalized data.


$$\text{CLR}\left(x_{i}\right)=log\left(\frac{x_{i}}{\text{geometric mean of all}\;x}\right)$$

Differences in labeling patterns between experimental conditions were tested using a permutational multivariate analysis of variance (PERMANOVA) using the vegan R package (114). Analysis was performed with 4,999 permutations, and *P* values were adjusted for multiple comparisons using the Benjamini-Hochberg (BH) procedure. Significantly different metabolites were considered those with an FDR-adjusted *P* value <0.01 and an effect size of >0.65. Significantly different metabolites were then included in pathway analysis that was performed as described above. Labeling extent (LE) (1-M0 fraction) was calculated for each metabolite (115) and used to generate principal components analysis scores plots with 95% confidence ellipses. For the clustered heatmap of glucose and glutamine, LE was transformed into z-scores to represent the range of incorporation more effectively. Clustered heatmaps were then generated using the pheatmap package in R using Euclidean distance hierarchical clustering at both the sample and metabolite level. For pathway heatmaps, KEGG compound IDs were used to map metabolic pathways associated with each metabolite. The average LE across each condition was calculated, and heatmaps were then generated for the listed pathways.

## Data Availability

The mass spectrometry proteomics data have been deposited to the ProteomeXchange Consortium via the PRIDE partner repository (https://www.ebi.ac.uk/pride/) with the data set identifier PXD066828. Raw metabolomics MS data are available at the MetaboLights (https://www.ebi.ac.uk/metabolights/) data repository under project accessions MTBLS12831 and MTBLS13492. RNAseq raw data available at EBI ENA (https://www.ebi.ac.uk/ena/browser/home) under projects PRJEB67890 and PRJEB83013.

## SUPPLEMENTAL MATERIAL

Supplemental figures mbio.03788-25-s0001.pdf Figures S1 to S5.

Table S1 mbio.03788-25-s0002.xlsx Summary data from global proteomics from untreated and ID parasites.

Table S2 mbio.03788-25-s0003.xlsx DISCO analysis of correlation between changes in iron depletion between proteomics and RNA-seq.

Table S3 mbio.03788-25-s0004.xlsx Summary data from metabolomics, including raw and normalized abundance data for identified metabolites.

Table S4 mbio.03788-25-s0005.xlsx Stable isotope labeling metabolomics data including the raw abundance data for each putative metabolite and associated isotopomers.

Table S5 mbio.03788-25-s0006.xlsx Stable isotope labeling metabolomics data with the normalized relative abundance data for both glucose and glutamine labeled metabolites.

ASM does not own the copyrights to Supplemental Material that may be linked to, or accessed through, an article. The authors have granted ASM a non-exclusive, world-wide license to publish the Supplemental Material files. Please contact the corresponding author directly for reuse.
