# Supplementary material for: Global translational and metabolic remodeling during iron deprivation in Toxoplasma gondii
Source: mBio. 2026 Apr 2;17(5):e03788-25. doi: 10.1128/mbio.03788-25 (PMC13170339; doi:10.1128/mbio.03788-25)
Supplement: Supplemental figures — Figures S1 to S5. [file mbio.03788-25-s0001.pdf]

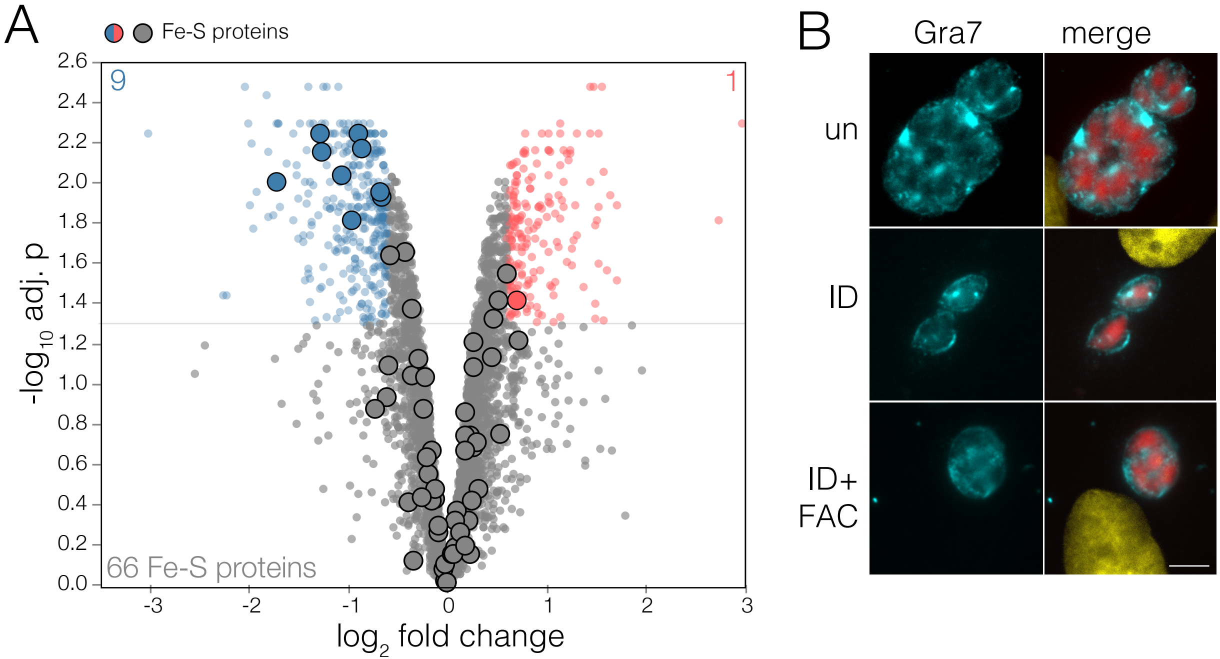

Figure S1. (**A**) Volcano plot of proteomics data from untreated and iron depleted parasites, highlighting significantly (adj. $p < 0.05$) upregulated (L2FC>0.6) (red) and downregulated (L2FC<-0.6) (blue) proteins. Each dot represents a protein with larger dots representing iron sulfur cluster (Fe-S) proteins (Renaud, Maupin and Besteiro, 2025) that appear in this dataset. (**B**) Immunofluorescence images showing Gra7 staining (teal) in untreated, iron depleted (ID) and iron complemented parasites (ID+FAC) (red). Gra7 visible inside parasites and on the parasitophorous vacuole, regardless of growth condition. Scale bar 5 µm.

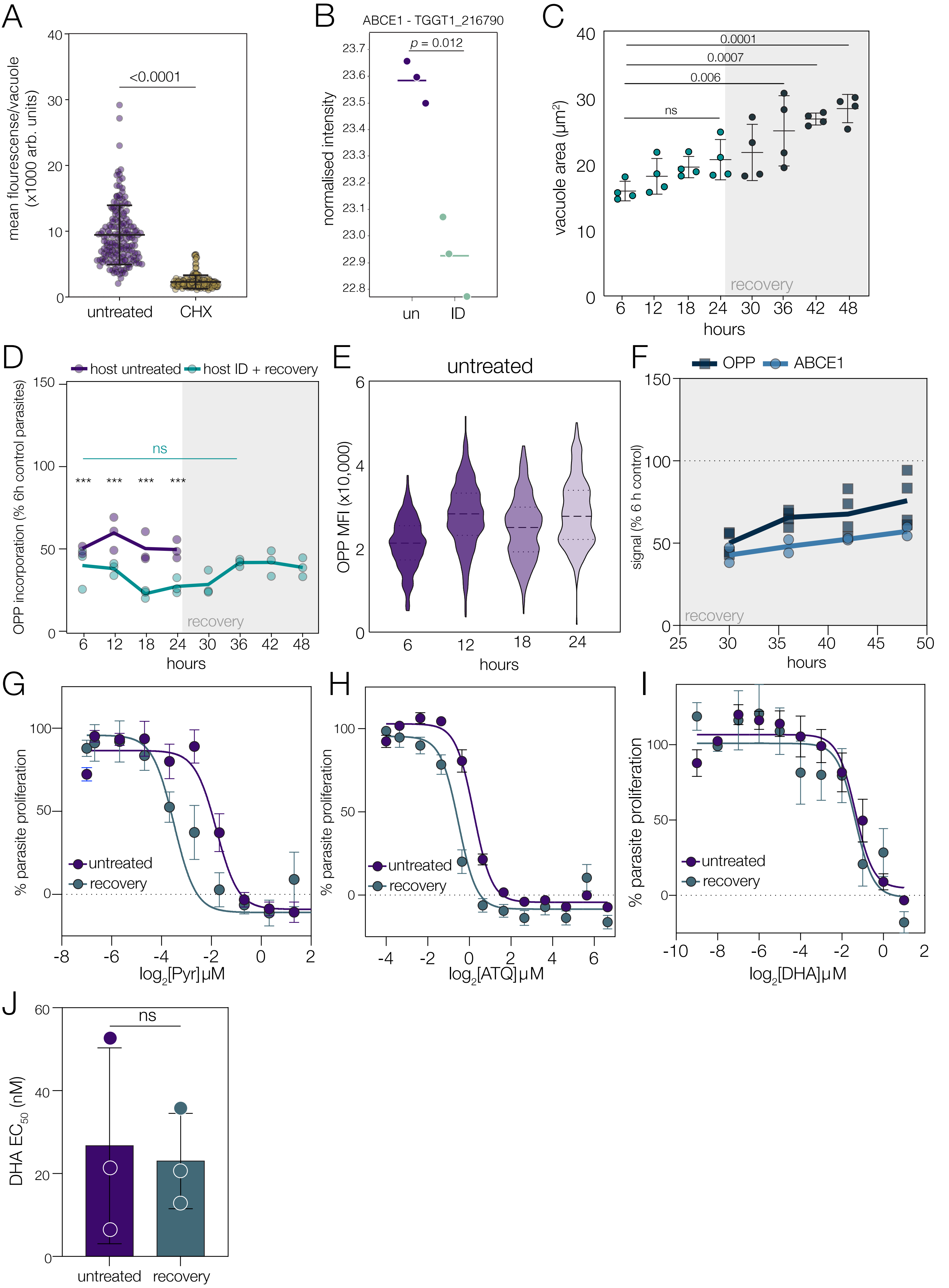

Figure S2. (**A**) Violin plot of mean OPP-incorporation in untreated or cycloheximide (CHX) treated parasites. Each point represents a parasite vacuole and bars at mean ±SD. *p* value from Welch's two tailed t-test (p<0.0001). (**B**) Plot of normalised (using variance stabilising normalisation (vsn)) ABCE1 protein intensities from MS in untreated and ID parasites. *p* values assigned by fitting a linear model. (**C**) Vacuole area measurements over time in ID and recovering parasites. Each point represents the mean vacuole size/replicate, bars at mean±SD. *p* values from one-way ANOVA with Tukey's correction. No significant differences were observed between timepoints for ID parasites. Compared to 6 hours of iron depletion, 12, 18 and 24 hours of recovery from iron deprivation coincided with a significant increase in vacuole size (p=0.006, p=0.0007 and p=0.0001 respectively). (**D**) HFF OPP incorporation. Each point represents the mean host cell incorporation from 5 fields of view, line at mean, normalised to parasites at 6 hours. *** indicates significant (p < 0.0001) difference between untreated and ID at indicated timepoint. Ns indicates no significant difference between untreated at 6h and recovery at 36 h. *p* values from two way ANOVA with Tukey's correction. (**E**) Violin plots of OPP in parasite vacuoles. Results from 4 biological replicates for 6, 12, 18 and 24 hours (N=382, 408, 460 and 323). Dotted lines at median and quartiles. (**F**) OPP incorporation and ABCE1 abundance in vacuoles > 20.1 μm². (**G-I**) Fluorescent growth assays testing the efficacy of pyrimethamine (**G**), atovaquone (**H**) or dihydroartemisinin (**I**) on untreated parasites or those recovering from iron depletion. Recovery parasites were previously ID as described, then moved to standard media. (**J**) Bar graph showing EC_{50} of DHA against untreated or recovering parasites. Bars at mean±SD. *p* value from two-tailed paired t test.

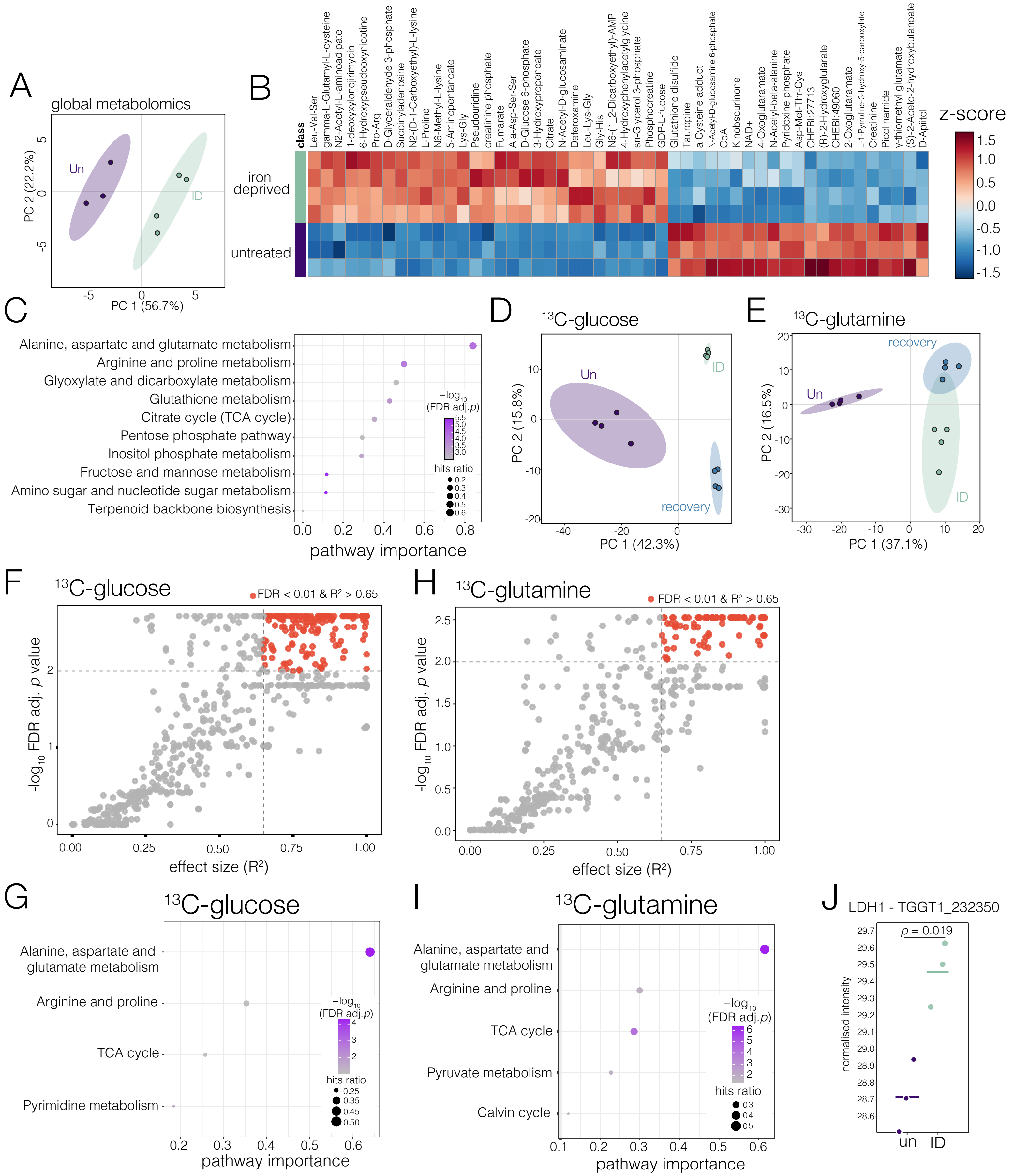

Figure S3 (A) PCA plot showing variation between replicates for untargeted metabolomics showing untreated and iron depleted conditions, ellipses represent the 95% confidence interval. (**B**) Heatmap of 50 most significantly different metabolites, clustered on Euclidian distance, coloured by z-score transformed fold change. (**C**) Dot plot of 10 most significantly enriched *Toxoplasma* KEGG pathways, ranked by pathway importance, computed from pathway topology analysis, with pathways containing closely related metabolites assigned greater importance. Dot size represents the proportion of metabolites for each term represented among significant metabolites and colour represents -log10(FDR adjusted *p* value). PCA plot showing variation between replicates across conditions for ^{13}C-glucose (**D**) and ^{13}C-glutamine (**E**) labelled metabolomics. Ellipses represent the 95% confidence interval. (**F**) Plot of PERMANOVA results testing differences in ^{13}C-glucose labelled isotopomer distributions. FDR adjusted *p* values are plotted against effect size (R2). Significantly different ($p = 0.01$ and $R^2 = 0.65$) metabolites are plotted in red, with 173 reaching the cutoff. (**G**) Dot plot of enriched *Toxoplasma* KEGG pathways within statistically different metabolites after ^{13}C-glucose labelling, ranked as above. Dot size represents the proportion of metabolites for each term represented among significant metabolites and colour represents -log10(FDR adjusted *p* value). (**H**) Plot of PERMANOVA results testing differences in ^{13}C-glutamine labelled isotopomer distributions. FDR adjusted *p* values are plotted against effect size (R^2). Significantly different ($p = 0.01$ and $R^2 = 0.65$) metabolites are plotted in red, with 91 reaching the cutoff. (**I**) Dot plot of enriched *Toxoplasma* KEGG pathways within statistically different metabolites after ^{13}C-glutamine labelling, ranked as above. (**J**) Plot of normalised (using variance stabilising normalisation (vsn)) LDH1 protein intensities from MS in untreated and ID parasites. *p* values from linear model.

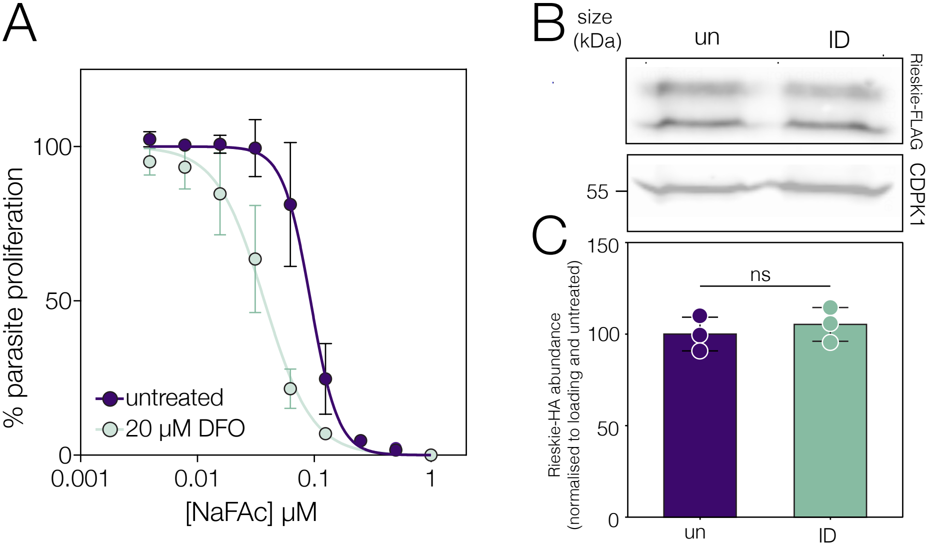

Figure S4. (**A**) Fluorescent parasite growth assay assessing the efficacy of aconitase inhibitor sodium fluoroacetate (NaFAc) against parasites cultured in full media and 20 μM DFO (N=4). (**B**) Immunoblot of lysates from Rieske-FLAG parasites, CDPK1 included as a loading control. (**C**) Quantification of Rieske-FLAG, points represent replicates (N=3), bar at mean±SD, *p* value from two-tailed paired t test.

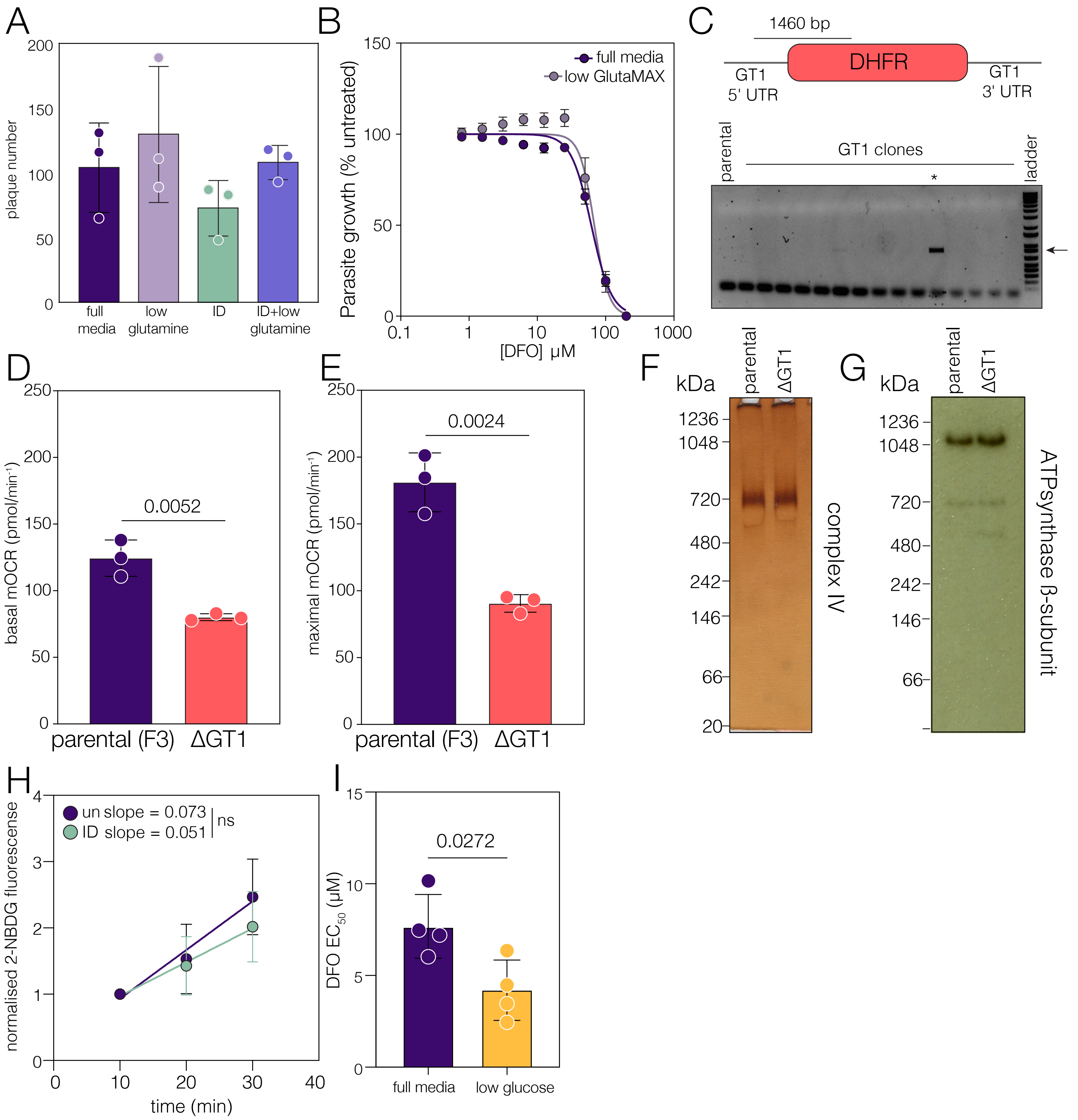

Figure S5. (**A**) Number of plaques counted for each media condition, bar at mean±SD . (**B**) Fluorescent parasite growth assay assessing the efficacy of DFO against parasites cultured in media with 4 mM GlutaMAX and low (0.4 mM) GlutaMAX media (N=3). (**C**) Diagram and PCR confirmation for ΔGT1 strain construction. (**D**) Basal and (**E**) maximal mOCR was determined for both parental and ΔGT1 parasite lines. Each point represents a replicate (N=3) *p* value from two-tailed paired t-tests .(**F**) Complex IV assay showing no change in complex IV activity between parental and ΔGT1 parasites. (**G**) Native western blot demonstrating ATPsynthase at correct size for full complex (probed using antibody against ATPsynthase ß-subunit.) (**H**) Normalised 2-NBDG fluorescence for untreated and iron depleted parasites was plotted for 10, 20 and 30 mins uptake, slope value calculated from linear regression, differenes tested using an extra sum of squares F-test. (**I**) EC_{50} for DFO in full media and low glucose media. *p* value from two-tailed t test.
